# Transcriptomic Analyses for Identification and Prioritization of Genes Associated With Alzheimer’s Disease in Humans

**DOI:** 10.3389/fbioe.2020.00031

**Published:** 2020-02-21

**Authors:** Yuchen Shi, Hui Liu, Changbo Yang, Kang Xu, Yangyang Cai, Zhao Wang, Zheng Zhao, Tingting Shao, Yixue Li

**Affiliations:** ^1^College of Bioinformatics Science and Technology, Harbin Medical University, Harbin, China; ^2^Beijing Neurosurgical Institute, Capital Medical University, Beijing, China

**Keywords:** long non-coding RNA, Alzheimer’s disease, transcriptomic analyses, RNA-seq, differential expression analysis

## Abstract

Long non-coding RNAs (lncRNAs), as important ncRNA regulators, play crucial roles in the regulation of various biological processes, and their aberrant expression is related to the occurrence and development of diseases, which is gradually validated by more and more studies. Alzheimer’s disease (AD) is a chronic neurodegenerative disease that often develops slowly and gradually deteriorates over time. However, which functions the lncRNAs perform in AD are almost unknown. In this study, we performed transcriptome analysis in AD, containing 12,892 known lncRNAs and 19,053 protein-coding genes (PCGs). Further, 14 down-regulated and 39 up-regulated lncRNAs were identified, compared with normal brain samples, which indicated that these lncRNAs might play critical roles in the pathogenesis of AD. In addition, 19 down-regulated and 28 up-regulated PCGs were also detected. Using the differentially expressed lncRNAs and PCGs through the WGCNA method, an lncRNA–mRNA co-expressed network was constructed. The results showed that lncRNAs RP3-522J7, MIR3180-2, and MIR3180-3 were frequently co-expressed with known AD risk PCGs. Interestingly, PCGs in the network are significantly enriched in brain- or AD-related biological functions, including the brain renin–angiotensin system, cell adhesion, neuroprotective role of THOP1 in AD, and so on. Furthermore, it was shown that 18 lncRNAs and 7 PCGs were highly expressed in normal brain tissue relative to other normal tissue types, suggesting their potential as diagnostic markers of AD, especially RP3-522J7, MIR3180-2, MIR3180-3, and CTA-929C8. In total, our study identified a compendium of AD-related dysregulated lncRNAs and characterized the corresponding biological functions of these lncRNAs in AD, which will be helpful to understand the molecular basis and pathogenesis of AD.

## Introduction

Alzheimer’s disease (AD) is an insidious progressive neurodegenerative disease, which is affected by many factors, including environmental factors and genetic and epigenetic variations (Toledo et al., 2015), but there is no doubt that aging is the biggest risk factor (Kold-Christensen and Johannsen, 2019). As the global population ages, there will be two billion people aged 60 years or over in the world by 2050 (Harper, 2014), and two new patients will be diagnosed with AD every minute, resulting in one million new patients who will be diagnosed every year (Alzheimer’s Association, 2015). Although the pathophysiology of AD still remains unclear (Xu et al., 2018), and even if there are no effective treatment methods currently, it is generally accepted that only the earliest intervention is likely to affect the progression of the disease (Sood et al., 2015).

With the development of life science research, it has been found that the human transcriptome is more complicated than previously known. Indeed, a considerable part of the human genome regions could transcribe as long non-coding RNAs (lncRNAs), a set of non-coding transcripts longer than 200 nt, which play important roles in various life activities, such as embryonic development, cell differentiation, aging, and complex diseases (Derrien et al., 2012; Rinn and Chang, 2012). Many studies have proved that lncRNAs are involved in many physiological and pathological processes through transcriptional or post-transcriptional regulatory mechanisms and thus play an important role in the process of the whole life, so it has become a hot spot of genetic and epigenetic research (Mercer and Mattick, 2013; Papait et al., 2013; Shi et al., 2013; Fatica and Bozzoni, 2014; Sultmann and Diederichs, 2014). As previous research has shown, due to the complex and transcriptional regulation mechanisms, lncRNAs could be focused on their roles in human complex diseases, including AD and cancers, to gain new insights into complex disease pathways, to identify biomarkers to improve diagnostic accuracy, and to examine the impact of treatment (Zhou et al., 2019). High-throughput sequencing technology has significant advantages, providing opportunities for insight into the genomic and transcriptomic research with large data sets, and it could be helpful to dissect the comprehensive transcriptome characterization of complex diseases (Sultan et al., 2008; Xiong et al., 2012; Prasad et al., 2016). However, the role of lncRNAs in the pathogenesis and progression of certain human complex diseases remains unclear, particularly AD. By comparing a ck-p25 AD model and control samples in mice, it was found that histone modification could regulate the differential expression of lncRNAs (Wan et al., 2016). Thus, it is indicated that lncRNAs might be helpful in investigating the transcription landscape of brain tissue in AD.

In this study, we aimed to identify the gene expression patterns in AD patients and controls, identify the lncRNAs involved in the AD-related dysregulated biological processes, and then to prioritize important lncRNAs based on the co-expression networks composed of differentially expressed lncRNAs and protein-coding genes (PCGs) in AD. Our results suggest that dysregulated expressed transcripts could affect complex disease, including lncRNAs and PCGs. Differentially expressed lncRNAs are significantly co-expressed with PCGs, which could be constructed in the co-expressed network to exhibit the complex regulatory relations in AD. Functional analysis revealed that lncRNAs might be involved in the AD-related biological processes, even regulate the AD-related function modules. Summarizing the above, our study provided a new set of AD-related dysregulated lncRNAs and identified the corresponding biological functions of these lncRNAs in AD. It will be helpful for further understanding the pathogenesis for AD.

## Materials and Methods

### RNA-seq Data Analysis

NGS data used in this research were derived from public resource GEO, including RNA-seq data of human cerebral cortex tissues from nine patients and eight controls (GEO accession GSE53697) (Scheckel et al., 2016). The RNA-seq data were mapped into the human reference genome (Hg38 version) by Tophat2 (Kim et al., 2013). For the measurement of expression values for lncRNAs and PCGs, we used Cufflinks v2.1.1 with FPKM (Trapnell et al., 2012).

### Differential Expression Analysis

Wilcoxon rank sum test was used to identify the differentially expressed lncRNAs and PCGs between the AD cases and the control group with the threshold of *P* < 0.01 (Han et al., 2019). Classification of lncRNAs and PCGs was according to the gene annotation file derived from GENCODE. The following biotypes are considered as known lncRNAs: “3prime_overlapping_ncrna,” “ambiguous_orf,” “antisense,” and “antisense_RNA,” “lincRNA,” “ncrna_host,” “non-_coding,” “non-_stop_decay,” “processed_transcript,” “retained_intron,” “sense_intronic,” and “sense_overlapping.” And the biotype of “protein_coding” is considered as a PCG.

### Collection of Aging-Related Gene Data Set

To investigate the relationship between differentially expressed genes in AD and aging, a list of aging-related genes was collected, which were defined as the genes involved in the development or aging-associated GO biological processes. And the functional annotation for genes and GO terms was derived from the Gene2GO file in NCBI^1^. And then, a cumulative hypergeometric test was used to investigate whether the AD-associated differentially expressed PCGs were enriched in the aging gene set with statistical significance.

### Co-expression Network Analysis

As important regulators, lncRNAs are considered to be involved the corresponding biological processes by regulating their target genes. For each differentially expressed lncRNA, we attempted to identify its regulatory target genes. The co-expression network for differentially expressed lncRNAs and PCGs was constructed by the WGCNA method with empirical threshold with the value equal to nine (Langfelder and Horvath, 2008).

### Gene Set Enrichment Analysis

To identify biological processes and cellular components which would be regulated by aberrantly expressed lncRNAs and PCGs in AD, enrichment analyses were performed for each lncRNA target PCG using the R package TCGAbiolinks (Colaprico et al., 2016), and GO/KEGG terms with adjusted *p* value < 0.01 by Benjamini–Hochberg methods were considered.

### Brain-Elevated Expression Analysis

The tissue-specific RNA-seq data set was obtained from the Genotype-Tissue Expression (GTEx, 2013) project, in which gene-level average RPKM values are reported for each tissue sample across 30 tissues including the brain (2013). A certain differentially expressed lncRNA or PCG would be defined as brain elevated if this transcription’s expression value in brain tissue is more than five times compared with the average value in all other tissues.

### AD-Related miRNA

To prioritize AD-related miRNA, we used the miRanda algorithm and searched the starBase database to obtain the mRNA–miRNA targeting relationship (Betel et al., 2010; Li et al., 2014). AD-related miRNAs were collected from the HMDD database, which manually curated the experimentally validated human disease-associated miRNA information (Huang et al., 2019). We analyzed the target relationship between AD-related miRNAs and differentially expressed PCGs and then ranked candidate miRNAs according to the count of the overlap between the differently expressed PCGs targeted by known AD miRNAs and the targets of each miRNA.

## Results

### Transcriptome Analysis in AD

**FIGURE 1:**
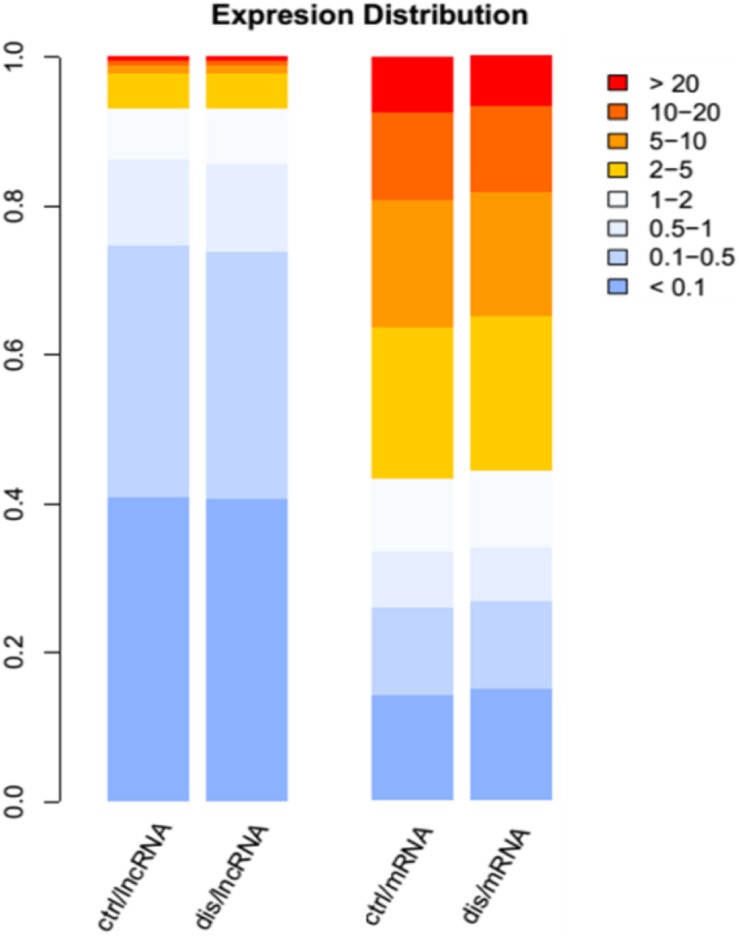
The distribution expression levels for long non-coding RNAs (lncRNAs) and mRNAs in Alzheimer’s disease (AD) and control groups. The different color legends represent the expression levels of lncRNAs and protein-coding genes (PCGs); the blue color represents low expression, and red color represents high expression.

A total of 17 RNA libraries were prepared from human brain samples, including nine AD and eight controls. From these samples, a total of 1,014,513,141 read pairs were generated from RNA-seq experiments, and >700 million read pairs (72.4%) were aligned to the human genome (Hg38 version). For the measurement of expression levels for lncRNAs and PCGs, we used Cufflinks. The gene expression levels were compared between AD and control samples, including lncRNAs and PCGs. The result revealed that about 12,892 lncRNAs and 19,053 PCGs were expressed. Consistently with previous studies, the expression levels of PCGs were generally higher than those of lncRNAs. In detail, there were just about 60% of lncRNAs with the FPKM value above 0.1, whereas the majority of PCGs were expressed above one (Figure 1). Moreover, 54.5% of lncRNAs were expressed in more than 15 samples and 1.6 times enriched for PCGs compared with lncRNAs (Supplementary Figure S1). These results revealed that lncRNAs were expressed widely in AD, indicating their potential important roles in the development of disease.

### Differential Expression of Both lncRNAs and PCGs in AD

**FIGURE 2:**
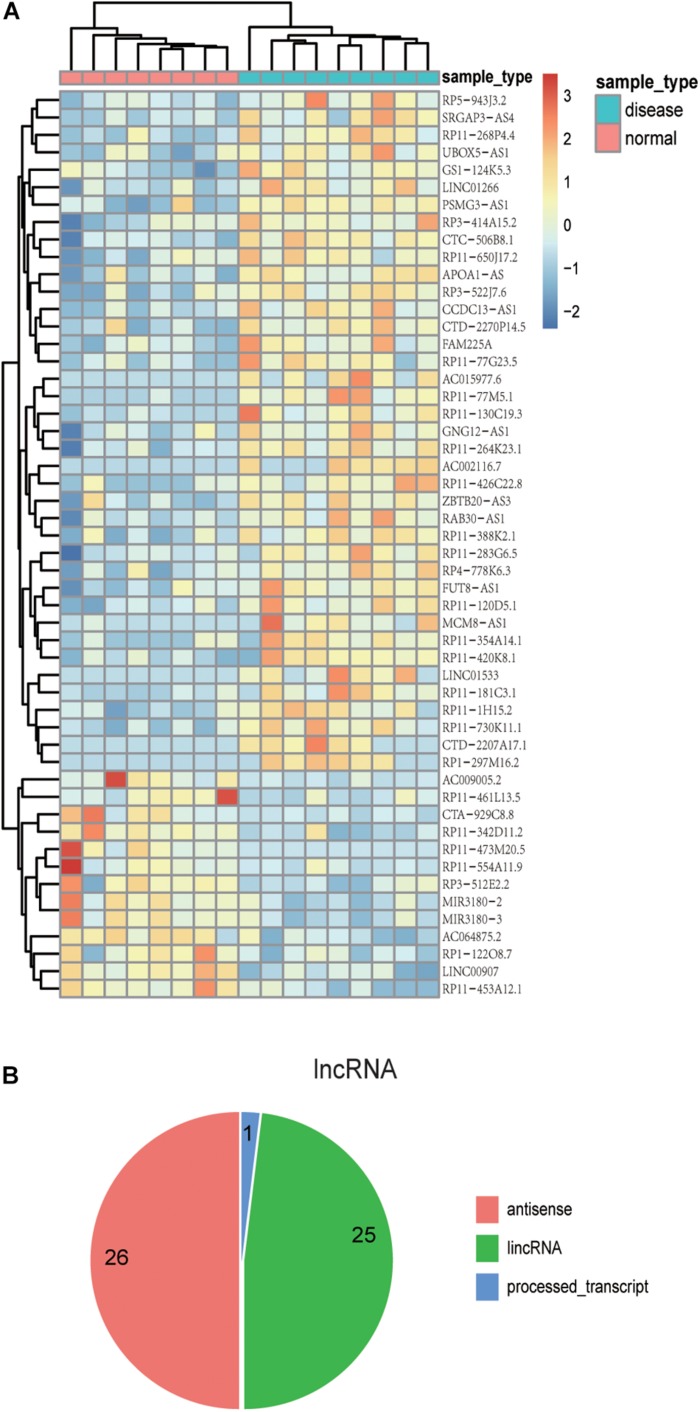
The expression patterns and localization of lncRNAs with differential expression. **(A)** The heat map shows the expression patterns of differentially expressed lncRNAs in both AD and control samples. Rows represent lncRNAs, and columns represent samples. Turquoise color in the top bar marks AD patients, and orange marks normal samples. **(B)** Pie chart displays distinct lncRNA classifications. The majority of lncRNAs are antisense lncRNAs or lincRNAs.

Previous studies have confirmed that the variant events of the human brain transcriptome are involved in the pathophysiological process of AD. Thus, the identification of the aberrantly expressed genes in AD patients might be helpful to understand the molecular mechanism of AD (Twine et al., 2011; Bernstein et al., 2016; Hensman Moss et al., 2017). We re-analyzed AD transcriptome data derived from a previous independent study which focused on ELAV-like protein binding to genes in the human brain yet did not analyze differential expression analysis of lncRNAs and PCGs (Scheckel et al., 2016). In this part, we removed the lncRNAs overlapping with PCGs in order to ensure the accuracy of the results. We found 100 differentially expressed genes, including 39 up-regulated lncRNAs and 14 down-regulated lncRNAs, as well as 28 up-regulated PCGs and 19 down-regulated PCGs (*p*-value < 0.01) (Supplementary Table S1). The unsupervised hierarchical clustering results revealed that differentially expressed lncRNAs (Figure 2A) and PCGs (Supplementary Figure S2) could well distinguish AD samples from control samples, indicating that there might be existing distinct signatures in expression level for AD. Notably, several lncRNAs, including CTA-929C8, RP11-461L13, and PSMG3-AS1, have relatively high expression levels in AD or control samples. For example, the expression value of CTA-929C8 in normal tissue is 0.3665 FPKM but decreases to 0.069 FPKM in AD. Another lncRNA is PSMG3-AS1, which is up-regulated with the mean expression value of 1.718 FPKM in AD.

Further, among the differentially expressed PCGs in AD, eight genes have been validated to be associated with AD, including VTI1A, CUX1, S100B, AGT, CD44, NTS, IRAK4, and AQP1, which are reported by the DisGeNET database (Pinero et al., 2017). The first two genes are down-regulated, and the others are up-regulated in AD. Moreover, VTI1A was validated to have susceptibility loci for late-onset AD (Grupe et al., 2006), even in glioma, based on a genome-wide association study (Kinnersley et al., 2015). As another example, CD44, a surface antigen expressed across multiple tissues, was up-regulated in AD samples, which corresponds with previous studies (Uberti et al., 2010). The results indicated that CD44 could play crucial roles in driving the immune response into infected tissues in the central nervous system. The aberrantly expressed S100B could seriously affect one of the hallmarks of AD and neuroinflammation; thus, it plays an important role in the pathophysiological process of AD, and the drugs targeting S100B might have a great impact on the treatment of this disease (Cirillo et al., 2015). These results indicate that the differentially expressed lncRNAs and PCGs are significantly associated with AD, which might affect the occurrence and development of AD.

Moreover, we investigated the genomic localization of differentially expressed lncRNAs compared with PCGs. As the results show in Figure 2B, 26 differentially expressed lncRNAs are antisense lncRNAs, and 25 are lincRNAs. Next, we computed the distance between differentially expressed lncRNAs and differentially expressed PCGs. As a result, we found that there are several lncRNAs located near important PCGs in the human genome. For example, six differentially expressed lncRNAs are located within 10 MB of S100B, and five of them are up-regulated in AD corresponding with S100B. The intergenic lncRNA, RP3-522J7, is an up-regulated lncRNA in AD with a fold change value of 2.65, compared with control samples, whose genomic distance is 3.3 MB apart from S100B. Therefore, it is concluded that the lncRNA PR3-522J7 might cis-regulate the expression of S100B. Another example is the gene CD44, which had 11 lncRNAs nearby, with a distance less than 10 Mb. Especially among the 11 neighbor lncRNAs, two lncRNAs described above, CTA-929C8 and CCDC13-AS1, are 7.8 and 7.5 MB apart from CD44. Therefore, we concluded that these two lncRNAs might cis-regulate the expression of CD44. These results indicate that some of these differentially expressed lncRNAs might cis-regulate the known AD protein-coding genes to influence the occurrence and development of AD.

Additionally, 92 development or age-related GO terms were collected to obtain a total of 2,435 age-related genes (Supplementary Table S2), and then, we found that 11 of these differentially expressed genes were identified as aging-related genes (Supplementary Table S3). The results of a cumulative hypergeometric test indicated that differentially expressed genes in diseases were significantly correlated with aging with statistical significance (*p* = 0.004650242), including AGT and CUX1, which are involved in function “aging” and “multicellular organism development,” respectively. Moreover, some differentially expressed genes are labeled as aging or longevity by the HAGR database and some studies. For example, S100B and AGT were labeled as aging genes in the HAGR database, while TOX3 was considered as a longevity gene (Tacutu et al., 2018). Another example is CLUAP1, which has been shown to vary significantly with age (Peters et al., 2015). These results indicate that these differentially expressed PCGs are associated with aging, confirm the effect of aging on AD, and are consistent with clinical observations.

### Co-expression Network Analysis Reveals Differentially Expressed Genes Involved in AD-Related Biological Functions

**FIGURE 3:**
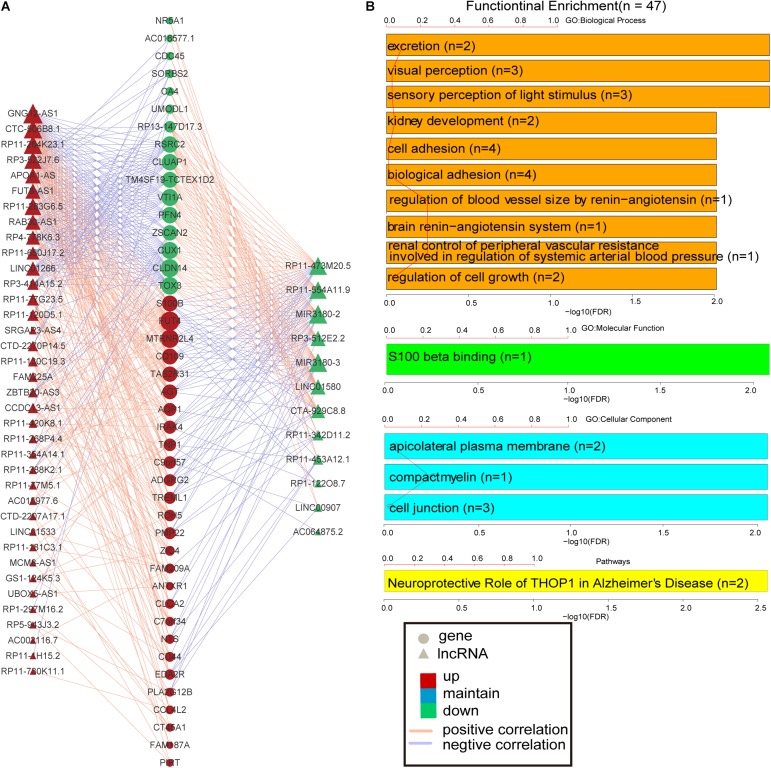
PCG–lncRNA co-expression network and function enrichment analysis. **(A)** The construction of PCG–lncRNA co-expression network. Circle represents gene, and triangle represents lncRNA. The orange line is positive correlation between lncRNA and PCG according to the Pearson correlation coefficient, and the blue color is negative. Red node marks up-regulated entity, and green node marks down-regulated entity. **(B)** Function enrichment analysis of the differentially expressed PCGs in the co-expression network.

More and more lncRNAs have been validated to play important roles in disease-associated biological processes (Sun et al., 2014; Zhou et al., 2017); however, there are still a considerable number of lncRNAs uncovered involved in the physiological process during embryonic development and cell differentiation, even in the pathological processes of human complex diseases. Based on the knowledge of the biological functions of the coding gene, the biological processes of lncRNAs could be predicted, with the hypothesis that if the expression of a certain lncRNA is correlated with the lists of PCGs, this gene co-expression pattern may provide reliable evidence that they are involved in the same or similar biological functions. The lncRNA–PCG co-expression network for differentially expressed lncRNAs and PCGs was constructed via R package WGCNA. As a result, the co-expression network was composed of 489 links between 49 differentially expressed lncRNAs and 44 PCGs (Figure 3A). These lncRNAs and PCGs are tightly connected together. Especially, S100B is co-expressed with 18 lncRNAs, including seven down-regulated lncRNAs and 11 up-regulated lncRNAs. For example, the weight value of the link between lncRNA RP3-522J7 and PCG S100B is 0.043867. Moreover, this lncRNA is located within 3.3 Mb from S100B. Therefore, we concluded that RP3-522J7 might cis-regulate the expression of S100B. In addition, we found that MIR3180-2 and MIR3180-3 shared relatively more co-expressed PCGs, including VTI1A, CUX1, S100B, AGT, NTS, and IRAK4, which are known as AD-related PCGs. Both the two lncRNAs are the host genes of hsa-miR-3180-3p and hsa-miR-3180-5p; thus, it was proposed that these two miRNAs might be up-regulated in AD.

**FIGURE 4:**
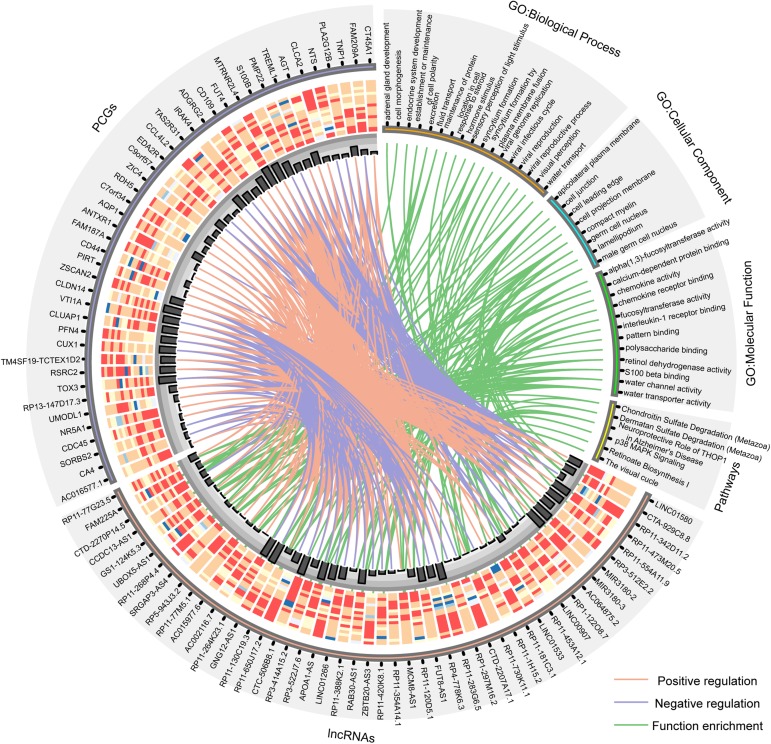
Circos plot shows the relationship between lncRNAs, targeted PCGs, and parts of functions (FDR < 0.005). The orange line is positive correlation between lncRNA and PCG according to the Pearson correlation coefficient, the blue color is negative, and the green lines connect lncRNAs and the functions that their targeted PCGs are enriched in. Bar plot shows the number of target relationships.

Furthermore, the GO and KEGG function enrichment analysis was performed for the differentially expressed PCGs in the co-expression network by the TCGAbiolinks tool (Xiong et al., 2012). Figure 3B and Supplementary Table S4 show the over-represented terms and pathways, among the most enriched of which are those involved in cell adhesion (FDR = 0.00969), regulation of cell growth (FDR = 0.00969), visual perception (FDR = 0.00460), brain renin–angiotensin system (FDR = 0.00969), neuroprotective role of THOP1 in AD (FDR = 0.00275), and so on. It’s worth noting that many PCGs targeted by differentially expressed lncRNAs in AD are involved in AD- and aging-related biological functions (Supplementary Table S5), such as CTA-929C8 target PCGs involved in the brain renin–angiotensin system (FDR = 0.00969), regulation of inflammatory response (FDR = 0.00969), and regulation of vasoconstriction (FDR = 0.00969) (Supplementary Figure S3); RP3-522J7.6 target PCGs involved in the brain renin–angiotensin system (FDR = 0.00944), the renal system process involved in the regulation of systemic arterial blood pressure (FDR = 0.00944), and the neurological system process involved in regulation of systemic arterial blood pressure (FDR = 0.00944) (Supplementary Figure S4); and MIR3180-2 and MIR3180-3 target PCGs involved in the neuroprotective role of THOP1 in AD (FDR = 0.00130) and blood vessel remodeling (FDR = 0.00944) (Supplementary Figures S5, S6). The relationship between lncRNAs, targeted PCGs, and parts of functions are shown in Figure 4 (FDR < 0.005). All these categories are associated with brain-related functions and are implicated in AD or aging. Therefore, these differentially expressed lncRNAs might be involved in AD and aging via regulating these molecular functions.

### Prioritization of Candidate lncRNAs as Potential Diagnostic Markers of AD

**FIGURE 5:**
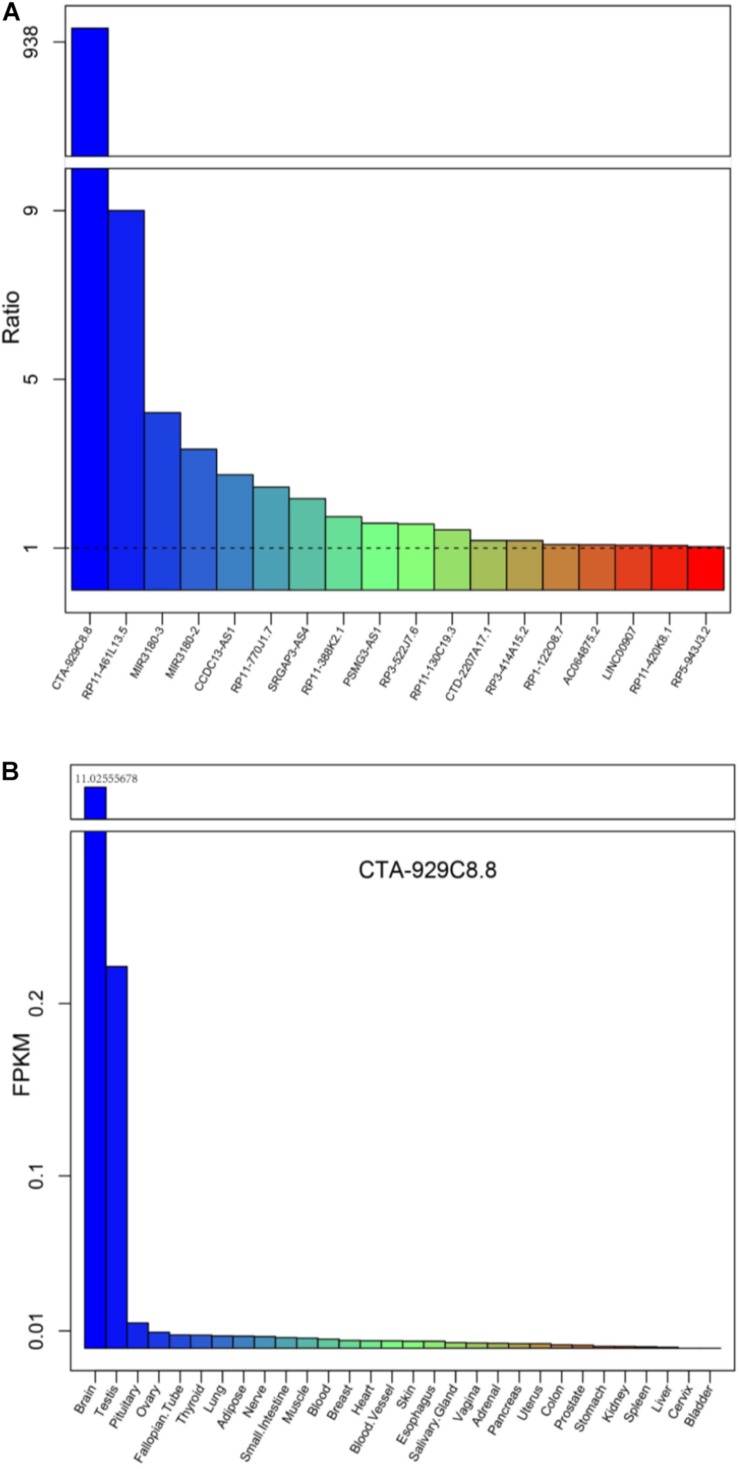
The mean expression levels of the differentially expressed lncRNAs for each tissue from the Genotype-Tissue Expression (GTEx) project. **(A)** The expression levels of the 18 lncRNAs up-regulated, expressed only in brain tissues, compared with the other tissue types are listed in the GTEx project. **(B)** The expression levels of a typical example CTA-929C8.8 among the 30 normal tissues in the GTEx project.

Further, we investigated whether these differentially expressed lncRNAs and PCGs tend to be particularly highly expressed in human cerebral tissues, which might be involved in brain-related biological processes and could be diagnostic markers of AD. We obtained high-throughput data from the GTEx project including 30 human tissues, and then the mean expression of the differentially expressed lncRNAs and PCGs was calculated (2013) (Figure 5A and Supplementary Figure S3). Eighteen differentially expressed lncRNAs are specifically highly expressed in cerebral tissue, including the lncRNAs described above, such as RP3-522J7, CCDC13-AS1, MIR3180-2, MIR3180-3, CTA-929C8, and so on (Figure 5A). On the other hand, there are seven PCGs, including three known AD-related genes, which are S100B, AGT, and NTS (Supplementary Figure S7). lncRNA CTA-929C8 is highly expressed in brain tissue, and its expression level was enriched in normal brain tissue, which was more than about 1,000-fold compared with other normal tissues (Figure 5B). We concluded that this lncRNA might be a suitable marker in AD. In addition, for the two PCGs, S100B and PIRT, we found that they are also enriched in normal brain tissues and that their expression levels were more than 10 times versus the other tissues (Supplementary Figure S7). Moreover, the two genes are up-regulated in AD. Therefore, it is indicated that the brain-specific highly expressed lncRNAs and PCGs might be involved in the brain-related biological processes, and even affect the brain-associated functions, and could be potential diagnostic markers for AD.

## Discussion

RNA-seq data of AD cortex tissue was obtained to identify the differentially expressed lncRNAs and PCGs; we established a co-expression network which could be helpful to identify a list of candidate AD-related lncRNAs. Further, these differentially expressed lncRNAs are cerebral tissue–specific, highly expressed in AD patients, and also co-expressed with AD-related PCGs. The lncRNA–PCG co-expressed network was constructed by WGCNA and was enriched in the AD-associated biological processes, such as cell adhesion, brain renin–angiotensin system, neuroprotective role of THOP1 in AD, and so on. Finally, several lncRNAs were identified, including MIR3180-2, MIR3180-3, and RP3-522J7, which were most highly co-expressed with the known AD-related genes in the network, such as S100B, AGT, NTS, and so on. These results suggested that the lncRNAs might directly regulate AD-related biological functions by targeting AD risk genes. Moreover, we further prioritized a set of differentially expressed lncRNAs and PCGs, which could be acting as potential AD markers, especially RP3-522J7, MIR3180-2, MIR3180-3, CTA-929C8, S100B, and PIRT. In summary, our study identified a compendium of lncRNAs and PCGs which have never been uncharacterized for AD and normal cerebral cortex tissue in previous research and could be prioritized as diagnostic markers for AD, which are helpful in identifying the high-risk population of AD, so as to intervene in prevention and treatment as early as possible.

Furthermore, the differentially expressed lncRNAs and PCGs identified in our study were validated by an independent data set (GEO accession GSE5281) (Liang et al., 2007). We found that 23 genes are uniformly differentially expressed. Especially, several key PCGs focused on by us are significantly differentially expressed in the test set, such as VTI1A, CUX1, S100B, AGT, CD44, and AQP1, and lncRNAs PSMG3-AS1 and FUT8-AS1. Webserver AlzData^2^ was also used to analyze AD-related PCG (Zhang et al., 2019). We found that the differently expressed genes are in conformity in different brain regions. And the result showed that S100B was differently expressed in the entorhinal cortex. Two PCGs, AGT and IRAK4, were expressed aberrantly in temporal cortex tissue. In addition, CD44 was differentially expressed in the entorhinal cortex, hippocampus, and temporal cortex, while another PCG AQP1 was differentially expressed in both the hippocampus and temporal cortex. These results suggested that AD-related genes may have a spatial–temporal expression pattern.

It is shown that the knowledge of the functions for the regulatory factors could be helpful for further understanding the occurrence and development of complex diseases, and the dysregulated miRNAs were validated to be associated with AD (Sadlon et al., 2019). Therefore, in our study, the AD-related miRNAs were further prioritized. By identifying the miRNA–mRNA targeting relationship using the miRanda algorithm, it was shown that 18 miRNAs regulating the differential expression of PCGs were labeled as AD-related miRNAs by HMDD, and the targeted PCGs contained seven AD-related PCGs, including VTI1A, CUX1, S100B, AGT, CD44, IRAK4, and AQP1. Then, based on the characteristics of known AD miRNAs, we ranked the miRNAs by the overlap between the differently expressed PCGs targeted by known AD miRNAs and the targets of each miRNA to provide clues for predicting AD miRNA candidates (Supplementary Table S6). The higher the rank of a certain miRNA is, the more it is possible for it to be considered as an AD-related miRNA. For example, as the second-ranked miRNA, Hsa-miR-1229 directly regulated the expression level of AD-related gene SORL1, and other targeted genes, which could be involved in the biological processes of nervous system development and neurological disease (Ghanbari et al., 2016). Moreover, this miRNA has been confirmed to dysregulate expression across the different brain tissue regions in AD patients (Puthiyedth et al., 2016). Hsa-mir-328 has been reported to be associated with a variety of neurologic disorders, such as autism spectrum disorder, Huntington’s, Parkinson’s, and Alzheimer’s, and the biological functions of its targeting genes APP and BACE1 were validated experimentally in mouse brain tissues (Boissonneault et al., 2009; Provost, 2010; Nt et al., 2018). High-throughput experimental data from starBase were used to verify the results, and similar conclusions were obtained (see Supplementary Text S1 and Supplementary Table S7). These results may be helpful for exploring the role of miRNAs in AD and providing novel insight for the study of the pathophysiology of AD.

## Data Availability Statement

The RNA-seq data used in this study were obtained from GSE53697 (https://www.ncbi.nlm.nih.gov/geo/query/acc.cgi?acc=GSE53697), GSE5281 (https://www.ncbi.nlm.nih.gov/geo/query/acc.cgi?acc=GSE5281), and GTEx (https://commonfund.nih.gov/GTEx/).

## Author Contributions

YL, TS, and ZZ conceived and designed the experiments. YS, HL, and CY performed the experiments and analyzed the data. YS, KX, YC, and ZW wrote the manuscript. All authors read and approved the final manuscript.

## Conflict of Interest

The authors declare that the research was conducted in the absence of any commercial or financial relationships that could be construed as a potential conflict of interest.

## Footnotes

1 https://www.ncbi.nlm.nih.gov/

2 www.alzdata.org
