# Supplementary material for: Transcriptomic Analyses for Identification and Prioritization of Genes Associated With Alzheimer’s Disease in Humans
Source: Front Bioeng Biotechnol. 2020 Feb 21;8:31. doi: 10.3389/fbioe.2020.00031 (PMC7047416; doi:10.3389/fbioe.2020.00031)
Supplement: Supplementary file 1 [file Data_Sheet_1.docx]

Supplementary Material

**Text S1**. Prioritization of AD-related miRNAs using data from starBase.

We also found that AD-related PCGs regulated by known AD-related miRNAs by exploring miRNA-target data obtained from the starBase database. Known AD miRNA targeting genes are significantly enriched in AD-related PCGs, such as VTI1A, CUX1, AGT, CD44, NTS, IRAK4 and AQP1. Then, we ranked the candidate miRNAs through the overlap of PCGs between AD-related miRNAs targeted and candidate miRNAs targeted (Table S7). Has-miR-519 was up-regulated expressed in the blood tissue of AD patients (Jia and Liu, 2016). And another miRNA Hsa-miR-545 provides well performance to distinguish AD samples form control samples. Thus, it might serve as a potential AD-related miRNA and its roles are worth to be further researched (Kumar et al., 2013; Cosin-Tomas et al., 2017)


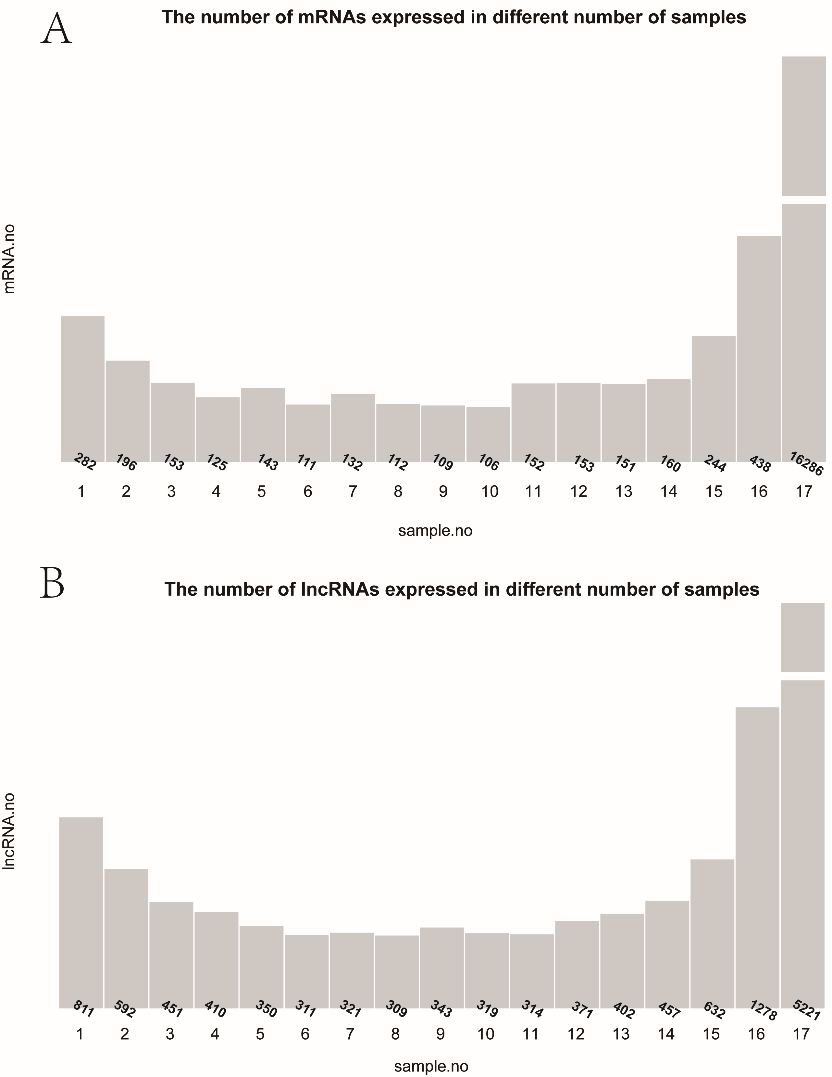


**Figure S1**. The number of lncRNAs (A) and mRNAs (B) expressed in different number of samples.


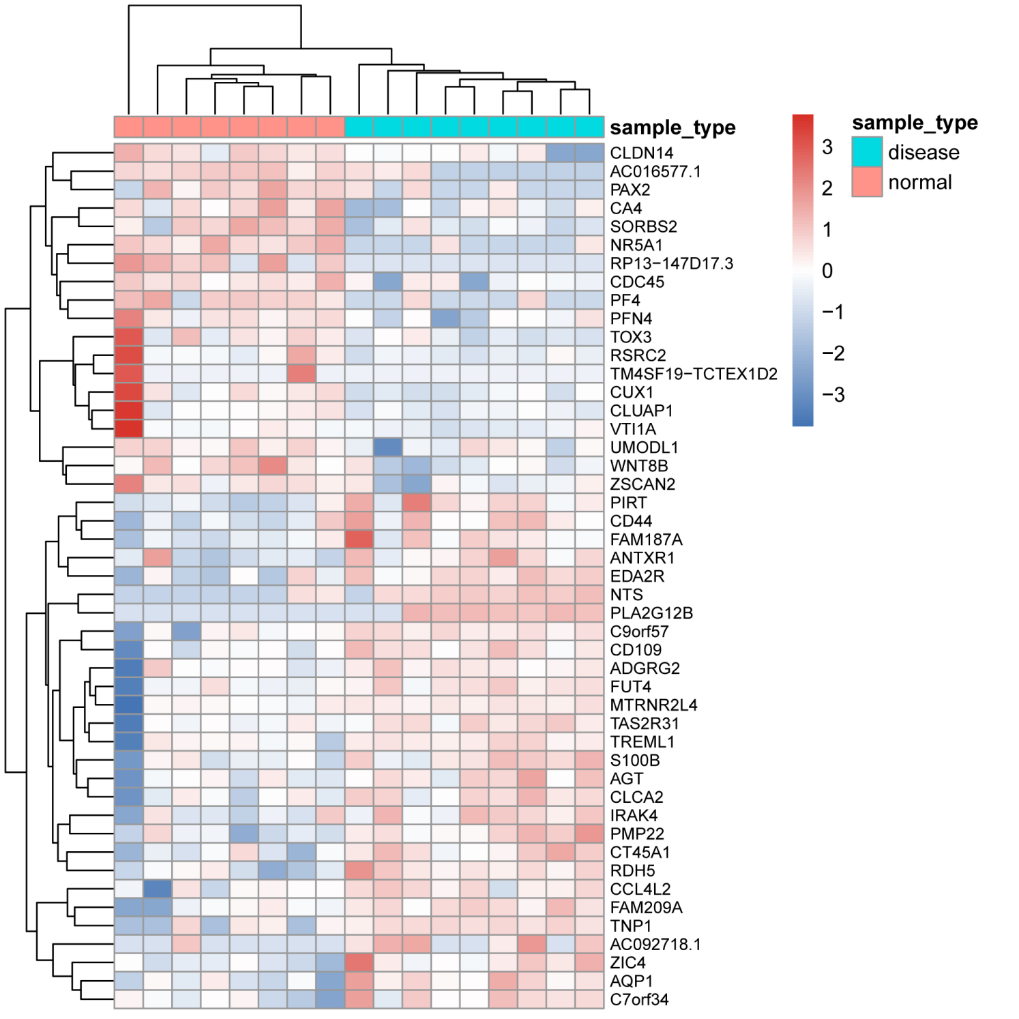


**Figure S2**. The heatmap shows the expression patterns of PCGs with differential expression in AD.


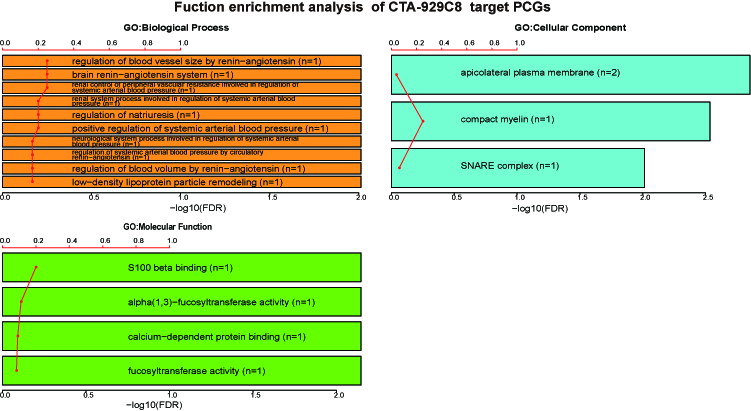


**Figure S3**. Function enrichment analysis of CTA-929C8 target PCGs.


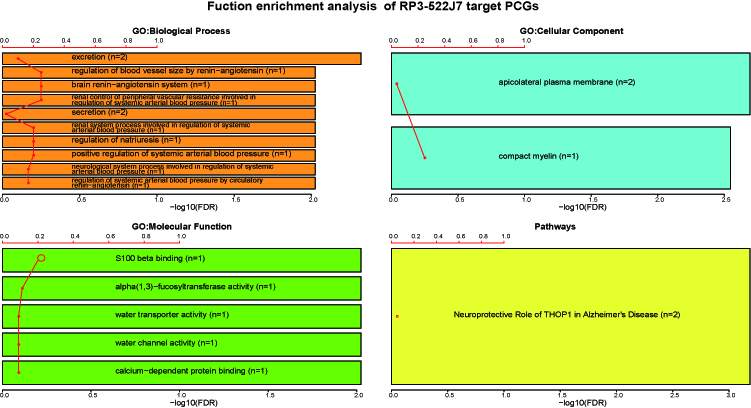


**Figure S4**. Function enrichment analysis of RP3-522J7target PCGs.


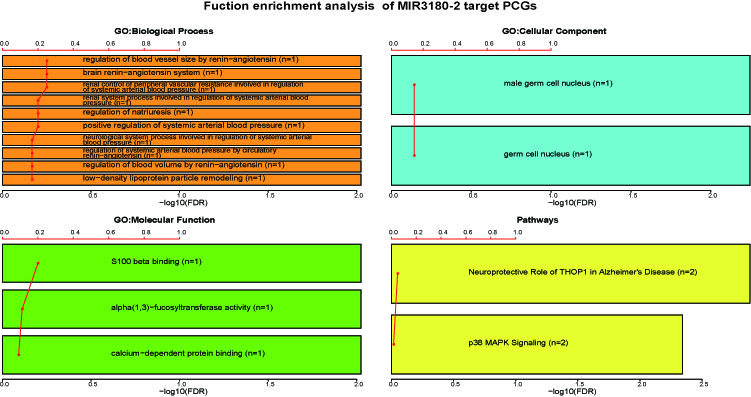


**Figure S5**. Function enrichment analysis of MIR3180-2 target PCGs.


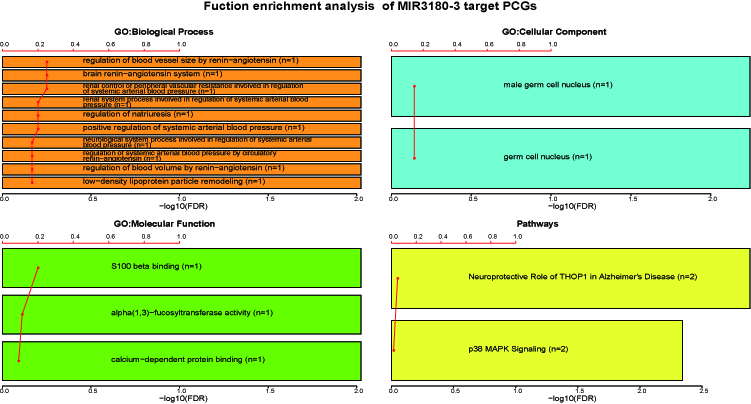


**Figure S6**. Function enrichment analysis of MIR3180-3 target PCGs.


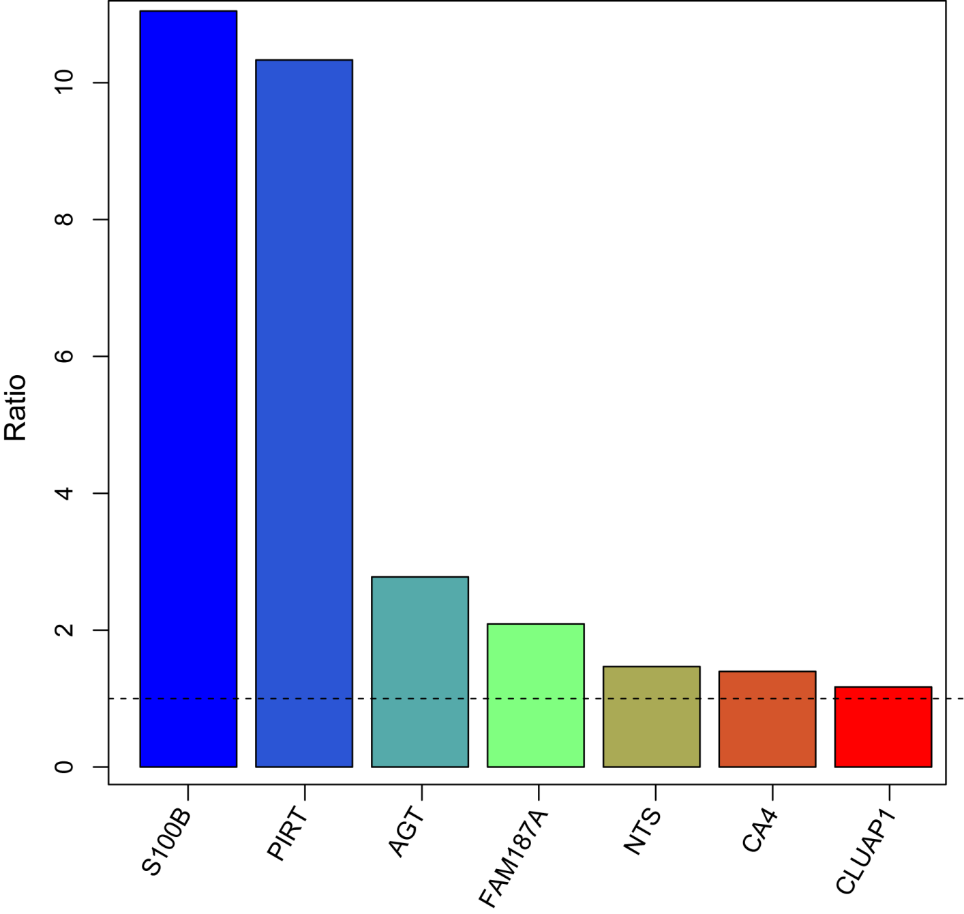


**Figure S7**. The mean expression of the differentially expressed lncRNAs for each tissue from GTEx project. Only PCGs with highly expressed in brain tissues relative to the other tissue types are listed.

**Table S1** Detailed information of differentiated lncRNAs and genes in network in Figure 3.

| node_name | node_type | node_expr | fc | mean_ctrl | mean_dis | p |
| --- | --- | --- | --- | --- | --- | --- |
| APOA1-AS | lnc | U | 1.672607 | 0.8780141 | 1.468572 | 0.003702 |
| CTD-2270P14.5 | lnc | U | 1.515118 | 0.4432909 | 0.671638 | 0.005512 |
| LINC00907 | lnc | D | 0.552332 | 0.4650841 | 0.256881 | 0.000329 |
| RP11-453A12.1 | lnc | D | 0.323533 | 0.0247366 | 0.008003 | 0.001181 |
| RP11-461L13.5 | lnc | D | 0.439505 | 0.33925 | 0.149102 | 0.007898 |
| RP11-77M5.1 | lnc | U | 18.00839 | 0.0103464 | 0.186323 | 0.000582 |
| RP11-1H15.2 | lnc | U | 1.98085 | 0.1984631 | 0.393126 | 0.002468 |
| CTA-929C8.8 | lnc | D | 0.188748 | 0.3653772 | 0.068964 | 0.006068 |
| FUT8-AS1 | lnc | U | 1.472428 | 0.4714851 | 0.694228 | 0.001563 |
| LINC01266 | lnc | U | 2.046654 | 0.1011826 | 0.207086 | 0.002468 |
| RP11-120D5.1 | lnc | U | 1.810772 | 0.1303507 | 0.236035 | 0.000987 |
| RP11-473M20.5 | lnc | D | 0.054197 | 0.0184513 | 0.001 | 0.000839 |
| RP11-554A11.9 | lnc | D | 0.408791 | 0.6779468 | 0.277138 | 0.007898 |
| RP3-522J7.6 | lnc | U | 2.648626 | 0.0279801 | 0.074109 | 0.005975 |
| MIR3180-2 | lnc | D | 0.438361 | 0.0219025 | 0.009601 | 0.001563 |
| MIR3180-3 | lnc | D | 0.438361 | 0.0219025 | 0.009601 | 0.001563 |
| RP11-264K23.1 | lnc | U | 1.922167 | 0.164482 | 0.316162 | 0.000576 |
| RP11-283G6.5 | lnc | U | 1.60817 | 1.7774884 | 2.858503 | 0.007898 |
| RP11-354A14.1 | lnc | U | 3.691567 | 0.0284096 | 0.104876 | 0.007915 |
| UBOX5-AS1 | lnc | U | 1.898738 | 0.1054966 | 0.20031 | 0.007898 |
| AC015977.6 | lnc | U | 45.10731 | 0.001 | 0.045107 | 0.008147 |
| GNG12-AS1 | lnc | U | 1.637834 | 0.489757 | 0.802141 | 0.003702 |
| RP11-77G23.5 | lnc | U | 3.476848 | 0.0263528 | 0.091625 | 0.007379 |
| CCDC13-AS1 | lnc | U | 1.699941 | 0.359131 | 0.610501 | 0.005512 |
| CTC-506B8.1 | lnc | U | 2.057567 | 0.3686478 | 0.758517 | 0.000329 |
| FAM225A | lnc | U | 1.654977 | 0.1432742 | 0.237115 | 0.007898 |
| RP11-130C19.3 | lnc | U | 3.877877 | 0.0855664 | 0.331816 | 0.000329 |
| RP11-268P4.4 | lnc | U | 3.831208 | 0.0230831 | 0.088436 | 0.003147 |
| RP11-342D11.2 | lnc | D | 0.633746 | 1.4498188 | 0.918817 | 0.003702 |
| RP11-650J17.2 | lnc | U | 1.992811 | 0.5040959 | 1.004568 | 0.007898 |
| SRGAP3-AS4 | lnc | U | 4.42799 | 0.0564916 | 0.250144 | 0.009829 |
| GS1-124K5.3 | lnc | U | 1.520511 | 0.8526854 | 1.296517 | 0.003702 |
| LINC01580 | lnc | D | 0.294759 | 0.0651823 | 0.019213 | 0.001099 |
| ZBTB20-AS3 | lnc | U | 2.026355 | 0.1640606 | 0.332445 | 0.007898 |
| MCM8-AS1 | lnc | U | 12.66401 | 0.0037966 | 0.048081 | 0.005762 |
| RP11-730K11.1 | lnc | U | 2.485217 | 0.1439593 | 0.35777 | 0.000576 |
| RP3-512E2.2 | lnc | D | 0.524302 | 0.4755262 | 0.24932 | 0.007898 |
| RP4-778K6.3 | lnc | U | 1.510946 | 0.5154953 | 0.778885 | 0.007898 |
| CTD-2207A17.1 | lnc | U | 8.341137 | 0.001 | 0.008341 | 0.008147 |
| RP3-414A15.2 | lnc | U | 1.761066 | 0.295468 | 0.520339 | 0.005512 |
| PSMG3-AS1 | lnc | U | 1.375196 | 1.2494011 | 1.718171 | 0.007898 |
| RAB30-AS1 | lnc | U | 1.407024 | 4.8445488 | 6.816398 | 0.001563 |
| RP11-388K2.1 | lnc | U | 2.674474 | 0.0623443 | 0.166738 | 0.007985 |
| LINC01533 | lnc | U | 36.30154 | 0.001 | 0.036302 | 0.00305 |
| RP11-181C3.1 | lnc | U | 4.998151 | 0.0597382 | 0.298581 | 0.003702 |
| RP11-420K8.1 | lnc | U | 2.709518 | 0.0774337 | 0.209808 | 0.005975 |
| RP1-297M16.2 | lnc | U | 26.0453 | 0.001 | 0.026045 | 0.008147 |
| AC002116.7 | lnc | U | 33.82412 | 0.001 | 0.033824 | 0.008147 |
| RP11-426C22.8 | lnc | U | 3.585344 | 0.0548621 | 0.1967 | 0.007379 |
| RP1-122O8.7 | lnc | D | 0.535048 | 0.5165438 | 0.276375 | 0.007898 |
| AC064875.2 | lnc | D | 0.613552 | 0.6616074 | 0.40593 | 0.002468 |
| RP5-943J3.2 | lnc | U | 2.49353 | 0.0338938 | 0.084515 | 0.008101 |
| AC009005.2 | lnc | D | 0.410877 | 0.2028821 | 0.08336 | 0.003702 |
| AC016577.1 | gene | D | 0.145939 | 0.364138 | 0.053142 | 0.001947 |
| AC092718.1 | gene | U | 10.55345 | 0.0082303 | 0.086858 | 0.005512 |
| ADGRG2 | gene | U | 1.408924 | 0.1414892 | 0.199348 | 0.005512 |
| AGT | gene | U | 1.408735 | 19.368463 | 27.28503 | 0.005512 |
| ANTXR1 | gene | U | 1.279985 | 3.66193 | 4.687217 | 0.007898 |
| AQP1 | gene | U | 1.851345 | 10.947936 | 20.26841 | 0.003702 |
| C7orf34 | gene | U | 1.836208 | 0.0999717 | 0.183569 | 0.007898 |
| C9orf57 | gene | U | 2.350494 | 0.0419172 | 0.098526 | 0.003318 |
| CA4 | gene | D | 0.7191 | 3.290815 | 2.366427 | 0.007898 |
| CCL4L2 | gene | U | 1.940292 | 0.0334651 | 0.064932 | 0.007898 |
| CD109 | gene | U | 1.590156 | 0.6678224 | 1.061942 | 0.005512 |
| CD44 | gene | U | 2.48715 | 4.94412 | 12.29677 | 0.003702 |
| CDC45 | gene | D | 0.251292 | 0.182809 | 0.045938 | 0.002422 |
| CLCA2 | gene | U | 3.254984 | 0.0385437 | 0.125459 | 0.002468 |
| CLDN14 | gene | D | 0.290948 | 0.0931206 | 0.027093 | 0.006068 |
| CLUAP1 | gene | D | 0.805121 | 9.7024588 | 7.811652 | 0.005512 |
| CT45A1 | gene | U | 4.161007 | 0.0095862 | 0.039888 | 0.006068 |
| CUX1 | gene | D | 0.740367 | 18.522838 | 13.7137 | 0.003702 |
| EDA2R | gene | U | 1.983733 | 0.1887904 | 0.37451 | 0.005512 |
| FAM187A | gene | U | 2.002252 | 0.8616491 | 1.725239 | 0.007898 |
| FAM209A | gene | U | 3.777125 | 0.0267699 | 0.101113 | 0.000576 |
| FUT4 | gene | U | 1.257263 | 0.5409816 | 0.680156 | 0.002468 |
| IRAK4 | gene | U | 1.295448 | 1.5091494 | 1.955024 | 0.007898 |
| MTRNR2L4 | gene | U | 1.735599 | 0.1556673 | 0.270176 | 0.001563 |
| NR5A1 | gene | D | 0.146732 | 0.0180875 | 0.002654 | 0.001193 |
| NTS | gene | U | 6.885316 | 0.0060152 | 0.041416 | 0.002357 |
| PAX2 | gene | D | 0.222721 | 0.0163243 | 0.003636 | 0.008236 |
| PF4 | gene | D | 0.120954 | 0.0798492 | 0.009658 | 0.004445 |
| PFN4 | gene | D | 0.446821 | 0.4828476 | 0.215747 | 0.005512 |
| PIRT | gene | U | 2.531615 | 0.2467695 | 0.624725 | 0.001563 |
| PLA2G12B | gene | U | 19.92691 | 0.001 | 0.019927 | 0.00305 |
| PMP22 | gene | U | 1.363249 | 14.281034 | 19.46861 | 0.001563 |
| RDH5 | gene | U | 1.5316 | 0.9272583 | 1.420189 | 0.000576 |
| RP13-147D17.3 | gene | D | 0.179036 | 0.0055855 | 0.001 | 0.002848 |
| RSRC2 | gene | D | 0.771733 | 21.0455 | 16.24151 | 0.007898 |
| S100B | gene | U | 1.385544 | 59.654563 | 82.65404 | 0.007898 |
| SORBS2 | gene | D | 0.815113 | 19.275463 | 15.71169 | 0.007898 |
| TAS2R31 | gene | U | 1.459741 | 0.5348598 | 0.780757 | 0.003702 |
| TM4SF19-TCTEX1D2 | gene | D | 0.170302 | 0.0058719 | 0.001 | 0.005492 |
| TNP1 | gene | U | 3.200666 | 0.0719745 | 0.230366 | 0.005791 |
| TOX3 | gene | D | 0.685067 | 0.8487569 | 0.581455 | 0.007898 |
| TREML1 | gene | U | 2.110645 | 0.1293272 | 0.272964 | 0.003702 |
| UMODL1 | gene | D | 0.426185 | 0.1408152 | 0.060013 | 0.005512 |
| VTI1A | gene | D | 0.714988 | 20.239075 | 14.4707 | 0.007898 |
| WNT8B | gene | D | 0.568234 | 0.1484321 | 0.084344 | 0.002468 |
| ZIC4 | gene | U | 1.295772 | 1.2070535 | 1.564066 | 0.000987 |
| ZSCAN2 | gene | D | 0.751349 | 1.374665 | 1.032853 | 0.003702 |

**Table S2** Go terms related to aging or development.

| cell aging |
| --- |
| response to reactive oxygen species |
| response to oxidative stress |
| developmental process |
| biological_process |
| multicellular organism aging |
| animal organ senescence |
| modulation of age-related behavioral decline |
| positive regulation of cell aging |
| negative regulation of cell aging |
| cellular senescence |
| replicative senescence |
| multicellular organism development |
| determination of adult lifespan |
| animal organ development |
| heterochromatin |
| heterochromatin assembly |
| positive regulation of germinal center formation |
| positive regulation of tolerance induction |
| positive regulation of mitochondrial fusion |
| positive regulation of mammary gland epithelial cell proliferation |
| positive regulation of embryonic development |
| positive regulation of cell differentiation |
| positive regulation of epidermis development |
| positive regulation of isotype switching |

**Table S3** Ageing related differentially expression PCGs.

| gene | GO | HAGR_long | HAGR_age |
| --- | --- | --- | --- |
| TNP1 | 1 | NA | NA |
| ZSCAN2 | 1 | NA | NA |
| SORBS2 | 1 | NA | NA |
| TOX3 | NA | 1 | NA |
| S100B | NA | NA | 1 |
| CD44 | 1 | NA | NA |
| EDA2R | 1 | NA | NA |
| AGT | 1 | 1 | NA |
| FAM209A | 1 | NA | NA |
| CUX1 | 1 | NA | NA |
| NR5A1 | 1 | NA | NA |
| PAX2 | 1 | NA | NA |
| PF4 | 1 | NA | NA |

**Table S4** Detailed information of function enrichment analysis of all the PCGs in the co-expression network.

| BP | GO:0007588 | excretion | FDR= 0.00460 |
| --- | --- | --- | --- |
|  | GO:0007601 | visual perception | FDR= 0.00460 |
|  | GO:0050953 | sensory perception of light stimulus | FDR= 0.00460 |
|  | GO:0001822 | kidney development | FDR= 0.00969 |
|  | GO:0007155 | cell adhesion | FDR= 0.00969 |
|  | GO:0022610 | biological adhesion | FDR= 0.00969 |
|  | GO:0002034 | regulation of blood vessel size by renin-angiotensin | FDR= 0.00969 |
|  | GO:0002035 | brain renin-angiotensin system | FDR= 0.00969 |
|  | GO:0003072 | renal control of peripheral vascular resistance involved in regulation of systemic arterial blood pressure | FDR= 0.00969 |
|  | GO:0001558 | regulation of cell growth | FDR= 0.00969 |
|  | GO:0048545 | response to steroid hormone stimulus | FDR= 0.00969 |
|  | GO:0003071 | renal system process involved in regulation of systemic arterial blood pressure | FDR= 0.00969 |
|  | GO:0003078 | regulation of natriuresis | FDR= 0.00969 |
|  | GO:0003084 | positive regulation of systemic arterial blood pressure | FDR= 0.00969 |
|  | GO:0001976 | neurological system process involved in regulation of systemic arterial blood pressure | FDR= 0.00969 |
|  | GO:0001991 | regulation of systemic arterial blood pressure by circulatory renin-angiotensin | FDR= 0.00969 |
|  | GO:0002016 | regulation of blood volume by renin-angiotensin | FDR= 0.00969 |
|  | GO:0034374 | low-density lipoprotein particle remodeling | FDR= 0.00969 |
|  | GO:0042147 | retrograde transport, endosome to Golgi | FDR= 0.00969 |
|  | GO:0044062 | regulation of excretion | FDR= 0.00969 |
| CC | GO:0016327 | apicolateral plasma membrane | FDR= 0.00827 |
|  | GO:0043218 | compact myelin | FDR= 0.00827 |
|  | GO:0030054 | cell junction | FDR= 0.00827 |
| MF | GO:0048154 | S100 beta binding | FDR= 0.00776 |
| Pathway |  | Neuroprotective Role of THOP1 in Alzheimer's Disease; ; | FDR= 0.00275 |

**Table S5** Detailed information of function enrichment analysis of each lncRNA targeted PCGs.

| lncRNA | GO_term/Pathway | FDR | GO_type |
| --- | --- | --- | --- |
| AC015977.6 | GO:0006833 | 0.00576 | BP |
| AC015977.6 | GO:0042044 | 0.00576 | BP |
| AC015977.6 | GO:0007588 | 0.00663 | BP |
| AC015977.6 | GO:0048667 | 0.00816 | BP |
| AC015977.6 | GO:0000904 | 0.00816 | BP |
| AC015977.6 | GO:0048545 | 0.00985 | BP |
| AC015977.6 | GO:0007409 | 0.00985 | BP |
| AC064875.2 | GO:0045892 | 0.0098 | BP |
| AC064875.2 | GO:0007283 | 0.0098 | BP |
| AC064875.2 | GO:0048232 | 0.0098 | BP |
| AC064875.2 | GO:0016481 | 0.0098 | BP |
| AC064875.2 | GO:0045934 | 0.0098 | BP |
| AC064875.2 | GO:0051172 | 0.0098 | BP |
| AC064875.2 | GO:0010629 | 0.0098 | BP |
| AC064875.2 | GO:0006974 | 0.0098 | BP |
| AC064875.2 | GO:0034621 | 0.0098 | BP |
| AC064875.2 | GO:0051253 | 0.0098 | BP |
| AC064875.2 | GO:0010558 | 0.0098 | BP |
| AC064875.2 | GO:0019953 | 0.00993 | BP |
| APOA1-AS | GO:0007601 | 0.00686 | BP |
| APOA1-AS | GO:0050953 | 0.00686 | BP |
| APOA1-AS | GO:0042147 | 0.00875 | BP |
| APOA1-AS | GO:0006891 | 0.00875 | BP |
| APOA1-AS | GO:0019317 | 0.00875 | BP |
| APOA1-AS | GO:0042355 | 0.00875 | BP |
| APOA1-AS | GO:0042354 | 0.00875 | BP |
| APOA1-AS | GO:0006004 | 0.00974 | BP |
| CCDC13-AS1 | GO:0006833 | 0.00658 | BP |
| CCDC13-AS1 | GO:0042044 | 0.00658 | BP |
| CCDC13-AS1 | GO:0007588 | 0.00756 | BP |
| CTA-929C8.8 | GO:0002034 | 0.00969 | BP |
| CTA-929C8.8 | GO:0002035 | 0.00969 | BP |
| CTA-929C8.8 | GO:0003072 | 0.00969 | BP |
| CTA-929C8.8 | GO:0003071 | 0.00969 | BP |
| CTA-929C8.8 | GO:0003078 | 0.00969 | BP |
| CTA-929C8.8 | GO:0003084 | 0.00969 | BP |
| CTA-929C8.8 | GO:0001976 | 0.00969 | BP |
| CTA-929C8.8 | GO:0001991 | 0.00969 | BP |
| CTA-929C8.8 | GO:0002016 | 0.00969 | BP |
| CTA-929C8.8 | GO:0034374 | 0.00969 | BP |
| CTA-929C8.8 | GO:0042147 | 0.00969 | BP |
| CTA-929C8.8 | GO:0044062 | 0.00969 | BP |
| CTA-929C8.8 | GO:0003081 | 0.00969 | BP |
| CTA-929C8.8 | GO:0009409 | 0.00969 | BP |
| CTA-929C8.8 | GO:0045723 | 0.00969 | BP |
| CTA-929C8.8 | GO:0009651 | 0.00969 | BP |
| CTA-929C8.8 | GO:0010873 | 0.00969 | BP |
| CTA-929C8.8 | GO:0030004 | 0.00969 | BP |
| CTA-929C8.8 | GO:0003014 | 0.00969 | BP |
| CTA-929C8.8 | GO:0010594 | 0.00969 | BP |
| CTA-929C8.8 | GO:0010872 | 0.00969 | BP |
| CTA-929C8.8 | GO:0001974 | 0.00969 | BP |
| CTA-929C8.8 | GO:0006970 | 0.00969 | BP |
| CTA-929C8.8 | GO:0042304 | 0.00969 | BP |
| CTA-929C8.8 | GO:0045923 | 0.00969 | BP |
| CTA-929C8.8 | GO:0006891 | 0.00969 | BP |
| CTA-929C8.8 | GO:0019317 | 0.00969 | BP |
| CTA-929C8.8 | GO:0042355 | 0.00969 | BP |
| CTA-929C8.8 | GO:0045777 | 0.00969 | BP |
| CTA-929C8.8 | GO:0045940 | 0.00969 | BP |
| CTA-929C8.8 | GO:0046889 | 0.00969 | BP |
| CTA-929C8.8 | GO:0019229 | 0.00969 | BP |
| CTA-929C8.8 | GO:0042354 | 0.00969 | BP |
| CTA-929C8.8 | GO:0019218 | 0.00969 | BP |
| CTA-929C8.8 | GO:0034367 | 0.00969 | BP |
| CTA-929C8.8 | GO:0034368 | 0.00969 | BP |
| CTA-929C8.8 | GO:0034369 | 0.00969 | BP |
| CTA-929C8.8 | GO:0048771 | 0.00969 | BP |
| CTA-929C8.8 | GO:0003044 | 0.00969 | BP |
| CTA-929C8.8 | GO:0010743 | 0.00969 | BP |
| CTA-929C8.8 | GO:0042310 | 0.00969 | BP |
| CTA-929C8.8 | GO:0001990 | 0.00969 | BP |
| CTA-929C8.8 | GO:0006004 | 0.00969 | BP |
| CTA-929C8.8 | GO:0016197 | 0.00969 | BP |
| CTA-929C8.8 | GO:0019217 | 0.00969 | BP |
| CTA-929C8.8 | GO:0048839 | 0.00969 | BP |
| CTA-929C8.8 | GO:0050727 | 0.00969 | BP |
| CTA-929C8.8 | GO:0050886 | 0.00969 | BP |
| CTC-506B8.1 | GO:0007588 | 0.00609 | BP |
| CTC-506B8.1 | GO:0001558 | 0.00994 | BP |
| CTC-506B8.1 | GO:0002034 | 0.00994 | BP |
| CTC-506B8.1 | GO:0002035 | 0.00994 | BP |
| CTC-506B8.1 | GO:0003072 | 0.00994 | BP |
| CTC-506B8.1 | GO:0046903 | 0.00994 | BP |
| CTC-506B8.1 | GO:0007155 | 0.00994 | BP |
| CTC-506B8.1 | GO:0022610 | 0.00994 | BP |
| CTC-506B8.1 | GO:0003071 | 0.00994 | BP |
| CTC-506B8.1 | GO:0003078 | 0.00994 | BP |
| CTC-506B8.1 | GO:0003084 | 0.00994 | BP |
| CTC-506B8.1 | GO:0001976 | 0.00994 | BP |
| CTC-506B8.1 | GO:0001991 | 0.00994 | BP |
| CTC-506B8.1 | GO:0002016 | 0.00994 | BP |
| CTC-506B8.1 | GO:0034374 | 0.00994 | BP |
| CTC-506B8.1 | GO:0042147 | 0.00994 | BP |
| CTC-506B8.1 | GO:0044062 | 0.00994 | BP |
| CTC-506B8.1 | GO:0000768 | 0.00994 | BP |
| CTC-506B8.1 | GO:0003081 | 0.00994 | BP |
| CTC-506B8.1 | GO:0006949 | 0.00994 | BP |
| CTC-506B8.1 | GO:0009409 | 0.00994 | BP |
| CTC-506B8.1 | GO:0045723 | 0.00994 | BP |
| CTC-506B8.1 | GO:0009651 | 0.00994 | BP |
| CTC-506B8.1 | GO:0010873 | 0.00994 | BP |
| CTC-506B8.1 | GO:0030004 | 0.00994 | BP |
| CTC-506B8.1 | GO:0003014 | 0.00994 | BP |
| CTC-506B8.1 | GO:0010594 | 0.00994 | BP |
| CTC-506B8.1 | GO:0010872 | 0.00994 | BP |
| CTC-506B8.1 | GO:0001974 | 0.00994 | BP |
| CTC-506B8.1 | GO:0006970 | 0.00994 | BP |
| CTC-506B8.1 | GO:0042304 | 0.00994 | BP |
| CTC-506B8.1 | GO:0045923 | 0.00994 | BP |
| CTC-506B8.1 | GO:0006891 | 0.00994 | BP |
| CTC-506B8.1 | GO:0019317 | 0.00994 | BP |
| CTC-506B8.1 | GO:0042355 | 0.00994 | BP |
| CTC-506B8.1 | GO:0045777 | 0.00994 | BP |
| CTC-506B8.1 | GO:0045940 | 0.00994 | BP |
| CTC-506B8.1 | GO:0046889 | 0.00994 | BP |
| CTD-2207A17.1 | GO:0000768 | 0.00269 | BP |
| CTD-2207A17.1 | GO:0006949 | 0.00269 | BP |
| CTD-2207A17.1 | GO:0016477 | 0.0086 | BP |
| CTD-2207A17.1 | GO:0048870 | 0.0086 | BP |
| CTD-2207A17.1 | GO:0051674 | 0.0086 | BP |
| CTD-2207A17.1 | GO:0001558 | 0.00925 | BP |
| CTD-2270P14.5 | GO:0007601 | 0.000365 | BP |
| CTD-2270P14.5 | GO:0050953 | 0.000365 | BP |
| CTD-2270P14.5 | GO:0000902 | 0.000365 | BP |
| FAM225A | GO:0006833 | 0.00576 | BP |
| FAM225A | GO:0042044 | 0.00576 | BP |
| FAM225A | GO:0007588 | 0.00662 | BP |
| FUT8-AS1 | GO:0002034 | 0.00966 | BP |
| FUT8-AS1 | GO:0002035 | 0.00966 | BP |
| FUT8-AS1 | GO:0003072 | 0.00966 | BP |
| FUT8-AS1 | GO:0003071 | 0.00966 | BP |
| FUT8-AS1 | GO:0003078 | 0.00966 | BP |
| FUT8-AS1 | GO:0003084 | 0.00966 | BP |
| FUT8-AS1 | GO:0001976 | 0.00966 | BP |
| FUT8-AS1 | GO:0001991 | 0.00966 | BP |
| FUT8-AS1 | GO:0002016 | 0.00966 | BP |
| FUT8-AS1 | GO:0034374 | 0.00966 | BP |
| FUT8-AS1 | GO:0042147 | 0.00966 | BP |
| FUT8-AS1 | GO:0044062 | 0.00966 | BP |
| FUT8-AS1 | GO:0003081 | 0.00966 | BP |
| FUT8-AS1 | GO:0009409 | 0.00966 | BP |
| FUT8-AS1 | GO:0045723 | 0.00966 | BP |
| FUT8-AS1 | GO:0009651 | 0.00966 | BP |
| FUT8-AS1 | GO:0010873 | 0.00966 | BP |
| FUT8-AS1 | GO:0030004 | 0.00966 | BP |
| FUT8-AS1 | GO:0003014 | 0.00966 | BP |
| FUT8-AS1 | GO:0010594 | 0.00966 | BP |
| FUT8-AS1 | GO:0010872 | 0.00966 | BP |
| FUT8-AS1 | GO:0001974 | 0.00966 | BP |
| FUT8-AS1 | GO:0006970 | 0.00966 | BP |
| FUT8-AS1 | GO:0019079 | 0.00966 | BP |
| FUT8-AS1 | GO:0042304 | 0.00966 | BP |
| FUT8-AS1 | GO:0045923 | 0.00966 | BP |
| FUT8-AS1 | GO:0006891 | 0.00966 | BP |
| FUT8-AS1 | GO:0019317 | 0.00966 | BP |
| FUT8-AS1 | GO:0042355 | 0.00966 | BP |
| FUT8-AS1 | GO:0045777 | 0.00966 | BP |
| FUT8-AS1 | GO:0045940 | 0.00966 | BP |
| FUT8-AS1 | GO:0046889 | 0.00966 | BP |
| FUT8-AS1 | GO:0019229 | 0.00966 | BP |
| FUT8-AS1 | GO:0042354 | 0.00966 | BP |
| FUT8-AS1 | GO:0019953 | 0.00966 | BP |
| FUT8-AS1 | GO:0019218 | 0.00966 | BP |
| FUT8-AS1 | GO:0034367 | 0.00966 | BP |
| FUT8-AS1 | GO:0034368 | 0.00966 | BP |
| FUT8-AS1 | GO:0034369 | 0.00966 | BP |
| FUT8-AS1 | GO:0048771 | 0.00966 | BP |
| FUT8-AS1 | GO:0003044 | 0.00966 | BP |
| FUT8-AS1 | GO:0010743 | 0.00966 | BP |
| FUT8-AS1 | GO:0019058 | 0.00966 | BP |
| FUT8-AS1 | GO:0042310 | 0.00966 | BP |
| FUT8-AS1 | GO:0001990 | 0.00974 | BP |
| FUT8-AS1 | GO:0006004 | 0.00974 | BP |
| FUT8-AS1 | GO:0007163 | 0.00974 | BP |
| GNG12-AS1 | GO:0007588 | 0.00575 | BP |
| GNG12-AS1 | GO:0001558 | 0.00883 | BP |
| GNG12-AS1 | GO:0002034 | 0.00883 | BP |
| GNG12-AS1 | GO:0002035 | 0.00883 | BP |
| GNG12-AS1 | GO:0003072 | 0.00883 | BP |
| GNG12-AS1 | GO:0046903 | 0.00883 | BP |
| GNG12-AS1 | GO:0003071 | 0.00883 | BP |
| GNG12-AS1 | GO:0003078 | 0.00883 | BP |
| GNG12-AS1 | GO:0003084 | 0.00883 | BP |
| GNG12-AS1 | GO:0001976 | 0.00883 | BP |
| GNG12-AS1 | GO:0001991 | 0.00883 | BP |
| GNG12-AS1 | GO:0002016 | 0.00883 | BP |
| GNG12-AS1 | GO:0034374 | 0.00883 | BP |
| GNG12-AS1 | GO:0042147 | 0.00883 | BP |
| GNG12-AS1 | GO:0044062 | 0.00883 | BP |
| GNG12-AS1 | GO:0000902 | 0.00883 | BP |
| GNG12-AS1 | GO:0000768 | 0.00883 | BP |
| GNG12-AS1 | GO:0003081 | 0.00883 | BP |
| GNG12-AS1 | GO:0006949 | 0.00883 | BP |
| GNG12-AS1 | GO:0009409 | 0.00883 | BP |
| GNG12-AS1 | GO:0045723 | 0.00883 | BP |
| GNG12-AS1 | GO:0009651 | 0.00901 | BP |
| GNG12-AS1 | GO:0010873 | 0.00901 | BP |
| GNG12-AS1 | GO:0030004 | 0.00901 | BP |
| GNG12-AS1 | GO:0003014 | 0.00915 | BP |
| GNG12-AS1 | GO:0010594 | 0.00915 | BP |
| GNG12-AS1 | GO:0010872 | 0.00915 | BP |
| GS1-124K5.3 | GO:0000768 | 0.00269 | BP |
| GS1-124K5.3 | GO:0006949 | 0.00269 | BP |
| GS1-124K5.3 | GO:0016477 | 0.0086 | BP |
| GS1-124K5.3 | GO:0048870 | 0.0086 | BP |
| GS1-124K5.3 | GO:0051674 | 0.0086 | BP |
| GS1-124K5.3 | GO:0001558 | 0.00925 | BP |
| LINC00907 | GO:0030325 | 0.00224 | BP |
| LINC00907 | GO:0032507 | 0.00224 | BP |
| LINC00907 | GO:0035270 | 0.00419 | BP |
| LINC01266 | GO:0042147 | 0.00895 | BP |
| LINC01266 | GO:0019079 | 0.00895 | BP |
| LINC01266 | GO:0006891 | 0.00895 | BP |
| LINC01266 | GO:0019317 | 0.00895 | BP |
| LINC01266 | GO:0042355 | 0.00895 | BP |
| LINC01266 | GO:0042354 | 0.00895 | BP |
| LINC01266 | GO:0019058 | 0.00895 | BP |
| LINC01266 | GO:0006004 | 0.00895 | BP |
| LINC01266 | GO:0007163 | 0.00895 | BP |
| LINC01266 | GO:0016197 | 0.00895 | BP |
| LINC01266 | GO:0048839 | 0.00895 | BP |
| LINC01580 | GO:0042147 | 0.00919 | BP |
| LINC01580 | GO:0006891 | 0.00919 | BP |
| LINC01580 | GO:0019317 | 0.00919 | BP |
| LINC01580 | GO:0042355 | 0.00919 | BP |
| LINC01580 | GO:0042354 | 0.00919 | BP |
| LINC01580 | GO:0006004 | 0.00974 | BP |
| MCM8-AS1 | GO:0019079 | 0.00466 | BP |
| MCM8-AS1 | GO:0019058 | 0.00466 | BP |
| MCM8-AS1 | GO:0007163 | 0.00466 | BP |
| MCM8-AS1 | GO:0019221 | 0.00513 | BP |
| MCM8-AS1 | GO:0001816 | 0.00513 | BP |
| MCM8-AS1 | GO:0022415 | 0.00513 | BP |
| MCM8-AS1 | GO:0016032 | 0.00513 | BP |
| MCM8-AS1 | GO:0006935 | 0.00874 | BP |
| MCM8-AS1 | GO:0042330 | 0.00874 | BP |
| MCM8-AS1 | GO:0007626 | 0.00956 | BP |
| MIR3180-2 | GO:0002034 | 0.00944 | BP |
| MIR3180-2 | GO:0002035 | 0.00944 | BP |
| MIR3180-2 | GO:0003072 | 0.00944 | BP |
| MIR3180-2 | GO:0003071 | 0.00944 | BP |
| MIR3180-2 | GO:0003078 | 0.00944 | BP |
| MIR3180-2 | GO:0003084 | 0.00944 | BP |
| MIR3180-2 | GO:0001976 | 0.00944 | BP |
| MIR3180-2 | GO:0001991 | 0.00944 | BP |
| MIR3180-2 | GO:0002016 | 0.00944 | BP |
| MIR3180-2 | GO:0034374 | 0.00944 | BP |
| MIR3180-2 | GO:0042147 | 0.00944 | BP |
| MIR3180-2 | GO:0044062 | 0.00944 | BP |
| MIR3180-2 | GO:0003081 | 0.00944 | BP |
| MIR3180-2 | GO:0009409 | 0.00944 | BP |
| MIR3180-2 | GO:0045723 | 0.00944 | BP |
| MIR3180-2 | GO:0009651 | 0.00944 | BP |
| MIR3180-2 | GO:0010873 | 0.00944 | BP |
| MIR3180-2 | GO:0030004 | 0.00944 | BP |
| MIR3180-2 | GO:0003014 | 0.00944 | BP |
| MIR3180-2 | GO:0010594 | 0.00944 | BP |
| MIR3180-2 | GO:0010872 | 0.00944 | BP |
| MIR3180-2 | GO:0001974 | 0.00944 | BP |
| MIR3180-2 | GO:0006970 | 0.00944 | BP |
| MIR3180-2 | GO:0042304 | 0.00944 | BP |
| MIR3180-2 | GO:0045923 | 0.00944 | BP |
| MIR3180-2 | GO:0006891 | 0.00944 | BP |
| MIR3180-2 | GO:0019317 | 0.00944 | BP |
| MIR3180-2 | GO:0042355 | 0.00944 | BP |
| MIR3180-2 | GO:0045777 | 0.00944 | BP |
| MIR3180-2 | GO:0045940 | 0.00944 | BP |
| MIR3180-2 | GO:0046889 | 0.00944 | BP |
| MIR3180-3 | GO:0002034 | 0.00944 | BP |
| MIR3180-3 | GO:0002035 | 0.00944 | BP |
| MIR3180-3 | GO:0003072 | 0.00944 | BP |
| MIR3180-3 | GO:0003071 | 0.00944 | BP |
| MIR3180-3 | GO:0003078 | 0.00944 | BP |
| MIR3180-3 | GO:0003084 | 0.00944 | BP |
| MIR3180-3 | GO:0001976 | 0.00944 | BP |
| MIR3180-3 | GO:0001991 | 0.00944 | BP |
| MIR3180-3 | GO:0002016 | 0.00944 | BP |
| MIR3180-3 | GO:0034374 | 0.00944 | BP |
| MIR3180-3 | GO:0042147 | 0.00944 | BP |
| MIR3180-3 | GO:0044062 | 0.00944 | BP |
| MIR3180-3 | GO:0003081 | 0.00944 | BP |
| MIR3180-3 | GO:0009409 | 0.00944 | BP |
| MIR3180-3 | GO:0045723 | 0.00944 | BP |
| MIR3180-3 | GO:0009651 | 0.00944 | BP |
| MIR3180-3 | GO:0010873 | 0.00944 | BP |
| MIR3180-3 | GO:0030004 | 0.00944 | BP |
| MIR3180-3 | GO:0003014 | 0.00944 | BP |
| MIR3180-3 | GO:0010594 | 0.00944 | BP |
| MIR3180-3 | GO:0010872 | 0.00944 | BP |
| MIR3180-3 | GO:0001974 | 0.00944 | BP |
| MIR3180-3 | GO:0006970 | 0.00944 | BP |
| MIR3180-3 | GO:0042304 | 0.00944 | BP |
| MIR3180-3 | GO:0045923 | 0.00944 | BP |
| MIR3180-3 | GO:0006891 | 0.00944 | BP |
| MIR3180-3 | GO:0019317 | 0.00944 | BP |
| MIR3180-3 | GO:0042355 | 0.00944 | BP |
| MIR3180-3 | GO:0045777 | 0.00944 | BP |
| MIR3180-3 | GO:0045940 | 0.00944 | BP |
| MIR3180-3 | GO:0046889 | 0.00944 | BP |
| RAB30-AS1 | GO:0002034 | 0.00945 | BP |
| RAB30-AS1 | GO:0002035 | 0.00945 | BP |
| RAB30-AS1 | GO:0003072 | 0.00945 | BP |
| RAB30-AS1 | GO:0003071 | 0.00945 | BP |
| RAB30-AS1 | GO:0003078 | 0.00945 | BP |
| RAB30-AS1 | GO:0003084 | 0.00945 | BP |
| RAB30-AS1 | GO:0001976 | 0.00945 | BP |
| RAB30-AS1 | GO:0001991 | 0.00945 | BP |
| RAB30-AS1 | GO:0002016 | 0.00945 | BP |
| RAB30-AS1 | GO:0034374 | 0.00945 | BP |
| RAB30-AS1 | GO:0042147 | 0.00945 | BP |
| RAB30-AS1 | GO:0044062 | 0.00945 | BP |
| RAB30-AS1 | GO:0003081 | 0.00945 | BP |
| RAB30-AS1 | GO:0009409 | 0.00945 | BP |
| RAB30-AS1 | GO:0045723 | 0.00945 | BP |
| RAB30-AS1 | GO:0009651 | 0.00945 | BP |
| RAB30-AS1 | GO:0010873 | 0.00945 | BP |
| RAB30-AS1 | GO:0030004 | 0.00945 | BP |
| RAB30-AS1 | GO:0003014 | 0.00945 | BP |
| RAB30-AS1 | GO:0010594 | 0.00945 | BP |
| RAB30-AS1 | GO:0010872 | 0.00945 | BP |
| RAB30-AS1 | GO:0001974 | 0.00945 | BP |
| RAB30-AS1 | GO:0006970 | 0.00945 | BP |
| RAB30-AS1 | GO:0042304 | 0.00945 | BP |
| RAB30-AS1 | GO:0045923 | 0.00945 | BP |
| RAB30-AS1 | GO:0006891 | 0.00945 | BP |
| RAB30-AS1 | GO:0019317 | 0.00945 | BP |
| RAB30-AS1 | GO:0042355 | 0.00945 | BP |
| RAB30-AS1 | GO:0045777 | 0.00945 | BP |
| RAB30-AS1 | GO:0045940 | 0.00945 | BP |
| RAB30-AS1 | GO:0046889 | 0.00945 | BP |
| RAB30-AS1 | GO:0019229 | 0.00945 | BP |
| RAB30-AS1 | GO:0042354 | 0.00945 | BP |
| RAB30-AS1 | GO:0019218 | 0.00945 | BP |
| RAB30-AS1 | GO:0034367 | 0.00945 | BP |
| RAB30-AS1 | GO:0034368 | 0.00945 | BP |
| RAB30-AS1 | GO:0034369 | 0.00945 | BP |
| RAB30-AS1 | GO:0048771 | 0.00945 | BP |
| RAB30-AS1 | GO:0003044 | 0.00949 | BP |
| RAB30-AS1 | GO:0010743 | 0.00949 | BP |
| RAB30-AS1 | GO:0042310 | 0.00949 | BP |
| RAB30-AS1 | GO:0001990 | 0.00974 | BP |
| RAB30-AS1 | GO:0006004 | 0.00974 | BP |
| RP1-122O8.7 | GO:0019317 | 0.00559 | BP |
| RP1-122O8.7 | GO:0042355 | 0.00559 | BP |
| RP1-122O8.7 | GO:0042354 | 0.00559 | BP |
| RP1-122O8.7 | GO:0006004 | 0.00559 | BP |
| RP1-122O8.7 | GO:0019221 | 0.00692 | BP |
| RP1-122O8.7 | GO:0001816 | 0.00692 | BP |
| RP1-122O8.7 | GO:0019320 | 0.00744 | BP |
| RP1-122O8.7 | GO:0046365 | 0.00744 | BP |
| RP1-122O8.7 | GO:0046164 | 0.00744 | BP |
| RP1-122O8.7 | GO:0044275 | 0.00782 | BP |
| RP1-122O8.7 | GO:0016052 | 0.00826 | BP |
| RP1-122O8.7 | GO:0009100 | 0.00882 | BP |
| RP1-122O8.7 | GO:0006486 | 0.00882 | BP |
| RP1-122O8.7 | GO:0043413 | 0.00882 | BP |
| RP1-122O8.7 | GO:0070085 | 0.00882 | BP |
| RP1-122O8.7 | GO:0009101 | 0.00926 | BP |
| RP11-120D5.1 | GO:0002034 | 0.00757 | BP |
| RP11-120D5.1 | GO:0002035 | 0.00757 | BP |
| RP11-120D5.1 | GO:0003072 | 0.00757 | BP |
| RP11-120D5.1 | GO:0003071 | 0.00757 | BP |
| RP11-120D5.1 | GO:0003078 | 0.00757 | BP |
| RP11-120D5.1 | GO:0003084 | 0.00757 | BP |
| RP11-120D5.1 | GO:0001976 | 0.00757 | BP |
| RP11-120D5.1 | GO:0001991 | 0.00757 | BP |
| RP11-120D5.1 | GO:0002016 | 0.00757 | BP |
| RP11-120D5.1 | GO:0034374 | 0.00757 | BP |
| RP11-120D5.1 | GO:0044062 | 0.00757 | BP |
| RP11-120D5.1 | GO:0003081 | 0.00757 | BP |
| RP11-120D5.1 | GO:0009409 | 0.00757 | BP |
| RP11-120D5.1 | GO:0045723 | 0.00757 | BP |
| RP11-120D5.1 | GO:0009651 | 0.00757 | BP |
| RP11-120D5.1 | GO:0010873 | 0.00757 | BP |
| RP11-120D5.1 | GO:0030004 | 0.00757 | BP |
| RP11-120D5.1 | GO:0003014 | 0.00757 | BP |
| RP11-120D5.1 | GO:0010594 | 0.00757 | BP |
| RP11-120D5.1 | GO:0010872 | 0.00757 | BP |
| RP11-120D5.1 | GO:0001974 | 0.00757 | BP |
| RP11-120D5.1 | GO:0006970 | 0.00757 | BP |
| RP11-120D5.1 | GO:0019079 | 0.00757 | BP |
| RP11-120D5.1 | GO:0042304 | 0.00757 | BP |
| RP11-120D5.1 | GO:0045923 | 0.00757 | BP |
| RP11-120D5.1 | GO:0019317 | 0.00757 | BP |
| RP11-120D5.1 | GO:0042355 | 0.00757 | BP |
| RP11-120D5.1 | GO:0045777 | 0.00757 | BP |
| RP11-120D5.1 | GO:0045940 | 0.00757 | BP |
| RP11-120D5.1 | GO:0046889 | 0.00757 | BP |
| RP11-120D5.1 | GO:0019229 | 0.00757 | BP |
| RP11-120D5.1 | GO:0042354 | 0.00757 | BP |
| RP11-120D5.1 | GO:0019218 | 0.00757 | BP |
| RP11-120D5.1 | GO:0034367 | 0.00757 | BP |
| RP11-120D5.1 | GO:0034368 | 0.00757 | BP |
| RP11-120D5.1 | GO:0034369 | 0.00757 | BP |
| RP11-120D5.1 | GO:0048771 | 0.00757 | BP |
| RP11-120D5.1 | GO:0003044 | 0.00757 | BP |
| RP11-120D5.1 | GO:0010743 | 0.00757 | BP |
| RP11-120D5.1 | GO:0019058 | 0.00757 | BP |
| RP11-120D5.1 | GO:0042310 | 0.00757 | BP |
| RP11-120D5.1 | GO:0001990 | 0.00757 | BP |
| RP11-120D5.1 | GO:0006004 | 0.00757 | BP |
| RP11-120D5.1 | GO:0007163 | 0.00757 | BP |
| RP11-120D5.1 | GO:0019217 | 0.00757 | BP |
| RP11-120D5.1 | GO:0050727 | 0.00757 | BP |
| RP11-120D5.1 | GO:0050886 | 0.00757 | BP |
| RP11-120D5.1 | GO:0003073 | 0.00757 | BP |
| RP11-120D5.1 | GO:0046890 | 0.00757 | BP |
| RP11-120D5.1 | GO:0051353 | 0.00757 | BP |
| RP11-120D5.1 | GO:0007155 | 0.00757 | BP |
| RP11-120D5.1 | GO:0022610 | 0.00757 | BP |
| RP11-120D5.1 | GO:0010565 | 0.00766 | BP |
| RP11-120D5.1 | GO:0007588 | 0.00825 | BP |
| RP11-120D5.1 | GO:0032844 | 0.00825 | BP |
| RP11-120D5.1 | GO:0045834 | 0.00838 | BP |
| RP11-120D5.1 | GO:0045926 | 0.00838 | BP |
| RP11-120D5.1 | GO:0016338 | 0.00905 | BP |
| RP11-120D5.1 | GO:0051341 | 0.00971 | BP |
| RP11-120D5.1 | GO:0055067 | 0.00994 | BP |
| RP11-130C19.3 | GO:0000768 | 0.00411 | BP |
| RP11-130C19.3 | GO:0006949 | 0.00411 | BP |
| RP11-130C19.3 | GO:0006833 | 0.00411 | BP |
| RP11-130C19.3 | GO:0042044 | 0.00411 | BP |
| RP11-130C19.3 | GO:0007588 | 0.00568 | BP |
| RP11-264K23.1 | GO:0007588 | 0.00476 | BP |
| RP11-264K23.1 | GO:0002034 | 0.00944 | BP |
| RP11-264K23.1 | GO:0002035 | 0.00944 | BP |
| RP11-264K23.1 | GO:0003072 | 0.00944 | BP |
| RP11-264K23.1 | GO:0046903 | 0.00944 | BP |
| RP11-264K23.1 | GO:0003071 | 0.00944 | BP |
| RP11-264K23.1 | GO:0003078 | 0.00944 | BP |
| RP11-264K23.1 | GO:0003084 | 0.00944 | BP |
| RP11-264K23.1 | GO:0001976 | 0.00944 | BP |
| RP11-264K23.1 | GO:0001991 | 0.00944 | BP |
| RP11-264K23.1 | GO:0002016 | 0.00944 | BP |
| RP11-264K23.1 | GO:0034374 | 0.00944 | BP |
| RP11-264K23.1 | GO:0042147 | 0.00944 | BP |
| RP11-264K23.1 | GO:0044062 | 0.00944 | BP |
| RP11-264K23.1 | GO:0003081 | 0.00944 | BP |
| RP11-264K23.1 | GO:0009409 | 0.00944 | BP |
| RP11-264K23.1 | GO:0045723 | 0.00944 | BP |
| RP11-264K23.1 | GO:0009651 | 0.00944 | BP |
| RP11-264K23.1 | GO:0010873 | 0.00944 | BP |
| RP11-264K23.1 | GO:0030004 | 0.00944 | BP |
| RP11-264K23.1 | GO:0003014 | 0.00944 | BP |
| RP11-264K23.1 | GO:0010594 | 0.00944 | BP |
| RP11-264K23.1 | GO:0010872 | 0.00944 | BP |
| RP11-264K23.1 | GO:0001974 | 0.00944 | BP |
| RP11-264K23.1 | GO:0006970 | 0.00944 | BP |
| RP11-264K23.1 | GO:0042304 | 0.00944 | BP |
| RP11-264K23.1 | GO:0045923 | 0.00944 | BP |
| RP11-264K23.1 | GO:0006891 | 0.00944 | BP |
| RP11-264K23.1 | GO:0019317 | 0.00944 | BP |
| RP11-264K23.1 | GO:0042355 | 0.00944 | BP |
| RP11-264K23.1 | GO:0045777 | 0.00944 | BP |
| RP11-264K23.1 | GO:0045940 | 0.00944 | BP |
| RP11-264K23.1 | GO:0046889 | 0.00944 | BP |
| RP11-268P4.4 | GO:0006833 | 0.00411 | BP |
| RP11-268P4.4 | GO:0042044 | 0.00411 | BP |
| RP11-268P4.4 | GO:0007588 | 0.00473 | BP |
| RP11-283G6.5 | GO:0002034 | 0.009 | BP |
| RP11-283G6.5 | GO:0002035 | 0.009 | BP |
| RP11-283G6.5 | GO:0003072 | 0.009 | BP |
| RP11-283G6.5 | GO:0003071 | 0.009 | BP |
| RP11-283G6.5 | GO:0003078 | 0.009 | BP |
| RP11-283G6.5 | GO:0003084 | 0.009 | BP |
| RP11-283G6.5 | GO:0001976 | 0.009 | BP |
| RP11-283G6.5 | GO:0001991 | 0.009 | BP |
| RP11-283G6.5 | GO:0002016 | 0.009 | BP |
| RP11-283G6.5 | GO:0034374 | 0.009 | BP |
| RP11-283G6.5 | GO:0042147 | 0.009 | BP |
| RP11-283G6.5 | GO:0044062 | 0.009 | BP |
| RP11-283G6.5 | GO:0003081 | 0.009 | BP |
| RP11-283G6.5 | GO:0009409 | 0.009 | BP |
| RP11-283G6.5 | GO:0045723 | 0.009 | BP |
| RP11-283G6.5 | GO:0009651 | 0.009 | BP |
| RP11-283G6.5 | GO:0010873 | 0.009 | BP |
| RP11-283G6.5 | GO:0030004 | 0.009 | BP |
| RP11-283G6.5 | GO:0003014 | 0.009 | BP |
| RP11-283G6.5 | GO:0010594 | 0.009 | BP |
| RP11-283G6.5 | GO:0010872 | 0.009 | BP |
| RP11-283G6.5 | GO:0001974 | 0.009 | BP |
| RP11-283G6.5 | GO:0006970 | 0.009 | BP |
| RP11-283G6.5 | GO:0042304 | 0.009 | BP |
| RP11-283G6.5 | GO:0045923 | 0.009 | BP |
| RP11-283G6.5 | GO:0006891 | 0.009 | BP |
| RP11-283G6.5 | GO:0019317 | 0.009 | BP |
| RP11-283G6.5 | GO:0042355 | 0.009 | BP |
| RP11-283G6.5 | GO:0045777 | 0.009 | BP |
| RP11-283G6.5 | GO:0045940 | 0.009 | BP |
| RP11-283G6.5 | GO:0046889 | 0.009 | BP |
| RP11-283G6.5 | GO:0019229 | 0.0093 | BP |
| RP11-283G6.5 | GO:0042354 | 0.0093 | BP |
| RP11-342D11.2 | GO:0048545 | 0.000861 | BP |
| RP11-342D11.2 | GO:0006833 | 0.005116 | BP |
| RP11-342D11.2 | GO:0042044 | 0.005116 | BP |
| RP11-342D11.2 | GO:0007588 | 0.00662 | BP |
| RP11-354A14.1 | GO:0002034 | 0.0054 | BP |
| RP11-354A14.1 | GO:0002035 | 0.0054 | BP |
| RP11-354A14.1 | GO:0003072 | 0.0054 | BP |
| RP11-354A14.1 | GO:0003071 | 0.0054 | BP |
| RP11-354A14.1 | GO:0003078 | 0.0054 | BP |
| RP11-354A14.1 | GO:0003084 | 0.0054 | BP |
| RP11-354A14.1 | GO:0001976 | 0.0054 | BP |
| RP11-354A14.1 | GO:0001991 | 0.0054 | BP |
| RP11-354A14.1 | GO:0002016 | 0.0054 | BP |
| RP11-354A14.1 | GO:0034374 | 0.0054 | BP |
| RP11-354A14.1 | GO:0044062 | 0.0054 | BP |
| RP11-354A14.1 | GO:0003081 | 0.0054 | BP |
| RP11-354A14.1 | GO:0009409 | 0.0054 | BP |
| RP11-354A14.1 | GO:0045723 | 0.0054 | BP |
| RP11-354A14.1 | GO:0009651 | 0.0054 | BP |
| RP11-354A14.1 | GO:0010873 | 0.0054 | BP |
| RP11-354A14.1 | GO:0030004 | 0.0054 | BP |
| RP11-354A14.1 | GO:0003014 | 0.0054 | BP |
| RP11-354A14.1 | GO:0010594 | 0.0054 | BP |
| RP11-354A14.1 | GO:0010872 | 0.0054 | BP |
| RP11-354A14.1 | GO:0001974 | 0.0054 | BP |
| RP11-354A14.1 | GO:0006970 | 0.0054 | BP |
| RP11-354A14.1 | GO:0042304 | 0.0054 | BP |
| RP11-354A14.1 | GO:0045923 | 0.0054 | BP |
| RP11-354A14.1 | GO:0045777 | 0.0054 | BP |
| RP11-354A14.1 | GO:0045940 | 0.0054 | BP |
| RP11-354A14.1 | GO:0046889 | 0.0054 | BP |
| RP11-354A14.1 | GO:0019229 | 0.0054 | BP |
| RP11-354A14.1 | GO:0019218 | 0.0054 | BP |
| RP11-354A14.1 | GO:0034367 | 0.0054 | BP |
| RP11-354A14.1 | GO:0034368 | 0.0054 | BP |
| RP11-354A14.1 | GO:0034369 | 0.0054 | BP |
| RP11-354A14.1 | GO:0048771 | 0.0054 | BP |
| RP11-354A14.1 | GO:0003044 | 0.0054 | BP |
| RP11-354A14.1 | GO:0010743 | 0.0054 | BP |
| RP11-354A14.1 | GO:0042310 | 0.0054 | BP |
| RP11-354A14.1 | GO:0001990 | 0.00556 | BP |
| RP11-354A14.1 | GO:0019217 | 0.00556 | BP |
| RP11-354A14.1 | GO:0050727 | 0.00556 | BP |
| RP11-354A14.1 | GO:0050886 | 0.00556 | BP |
| RP11-354A14.1 | GO:0003073 | 0.00556 | BP |
| RP11-354A14.1 | GO:0046890 | 0.00556 | BP |
| RP11-354A14.1 | GO:0051353 | 0.00556 | BP |
| RP11-354A14.1 | GO:0010565 | 0.00578 | BP |
| RP11-354A14.1 | GO:0007588 | 0.00618 | BP |
| RP11-354A14.1 | GO:0032844 | 0.00618 | BP |
| RP11-354A14.1 | GO:0045834 | 0.00623 | BP |
| RP11-354A14.1 | GO:0045926 | 0.00623 | BP |
| RP11-354A14.1 | GO:0051341 | 0.00732 | BP |
| RP11-354A14.1 | GO:0055067 | 0.00747 | BP |
| RP11-354A14.1 | GO:0050909 | 0.00791 | BP |
| RP11-354A14.1 | GO:0040014 | 0.008 | BP |
| RP11-354A14.1 | GO:0035150 | 0.008 | BP |
| RP11-354A14.1 | GO:0050880 | 0.008 | BP |
| RP11-354A14.1 | GO:0008217 | 0.008 | BP |
| RP11-354A14.1 | GO:0019216 | 0.008 | BP |
| RP11-354A14.1 | GO:0003018 | 0.00839 | BP |
| RP11-354A14.1 | GO:0048514 | 0.00878 | BP |
| RP11-354A14.1 | GO:0060191 | 0.00878 | BP |
| RP11-354A14.1 | GO:0001568 | 0.00878 | BP |
| RP11-354A14.1 | GO:0001944 | 0.00878 | BP |
| RP11-354A14.1 | GO:0051272 | 0.00878 | BP |
| RP11-354A14.1 | GO:0001822 | 0.00878 | BP |
| RP11-354A14.1 | GO:0003013 | 0.00878 | BP |
| RP11-354A14.1 | GO:0008015 | 0.00878 | BP |
| RP11-354A14.1 | GO:0008406 | 0.00878 | BP |
| RP11-354A14.1 | GO:0008585 | 0.00878 | BP |
| RP11-354A14.1 | GO:0040017 | 0.00878 | BP |
| RP11-354A14.1 | GO:0001655 | 0.00909 | BP |
| RP11-354A14.1 | GO:0030198 | 0.00933 | BP |
| RP11-354A14.1 | GO:0042445 | 0.00933 | BP |
| RP11-354A14.1 | GO:0045137 | 0.00933 | BP |
| RP11-354A14.1 | GO:0048608 | 0.0094 | BP |
| RP11-354A14.1 | GO:0007187 | 0.00988 | BP |
| RP11-354A14.1 | GO:0007548 | 0.00994 | BP |
| RP11-388K2.1 | GO:0019317 | 0.00524 | BP |
| RP11-388K2.1 | GO:0042355 | 0.00524 | BP |
| RP11-388K2.1 | GO:0042354 | 0.00524 | BP |
| RP11-388K2.1 | GO:0006004 | 0.00524 | BP |
| RP11-388K2.1 | GO:0016338 | 0.00658 | BP |
| RP11-388K2.1 | GO:0019320 | 0.00784 | BP |
| RP11-388K2.1 | GO:0046365 | 0.00784 | BP |
| RP11-388K2.1 | GO:0046164 | 0.00784 | BP |
| RP11-388K2.1 | GO:0044275 | 0.00813 | BP |
| RP11-388K2.1 | GO:0016052 | 0.00851 | BP |
| RP11-420K8.1 | GO:0019317 | 0.00785 | BP |
| RP11-420K8.1 | GO:0042355 | 0.00785 | BP |
| RP11-420K8.1 | GO:0042354 | 0.00785 | BP |
| RP11-420K8.1 | GO:0006004 | 0.00785 | BP |
| RP11-420K8.1 | GO:0019221 | 0.00864 | BP |
| RP11-420K8.1 | GO:0001816 | 0.00864 | BP |
| RP11-420K8.1 | GO:0050909 | 0.00864 | BP |
| RP11-420K8.1 | GO:0019320 | 0.00994 | BP |
| RP11-420K8.1 | GO:0046365 | 0.00994 | BP |
| RP11-453A12.1 | GO:0030325 | 0.00449 | BP |
| RP11-453A12.1 | GO:0032507 | 0.00449 | BP |
| RP11-453A12.1 | GO:0035270 | 0.00836 | BP |
| RP11-473M20.5 | GO:0002034 | 0.00953 | BP |
| RP11-473M20.5 | GO:0002035 | 0.00953 | BP |
| RP11-473M20.5 | GO:0003072 | 0.00953 | BP |
| RP11-473M20.5 | GO:0003071 | 0.00953 | BP |
| RP11-473M20.5 | GO:0003078 | 0.00953 | BP |
| RP11-473M20.5 | GO:0003084 | 0.00953 | BP |
| RP11-473M20.5 | GO:0001976 | 0.00953 | BP |
| RP11-473M20.5 | GO:0001991 | 0.00953 | BP |
| RP11-473M20.5 | GO:0002016 | 0.00953 | BP |
| RP11-473M20.5 | GO:0034374 | 0.00953 | BP |
| RP11-473M20.5 | GO:0042147 | 0.00953 | BP |
| RP11-473M20.5 | GO:0044062 | 0.00953 | BP |
| RP11-473M20.5 | GO:0003081 | 0.00953 | BP |
| RP11-473M20.5 | GO:0009409 | 0.00953 | BP |
| RP11-473M20.5 | GO:0045723 | 0.00953 | BP |
| RP11-473M20.5 | GO:0009651 | 0.00953 | BP |
| RP11-473M20.5 | GO:0010873 | 0.00953 | BP |
| RP11-473M20.5 | GO:0030004 | 0.00953 | BP |
| RP11-473M20.5 | GO:0003014 | 0.00953 | BP |
| RP11-473M20.5 | GO:0010594 | 0.00953 | BP |
| RP11-473M20.5 | GO:0010872 | 0.00953 | BP |
| RP11-473M20.5 | GO:0001974 | 0.00953 | BP |
| RP11-473M20.5 | GO:0006970 | 0.00953 | BP |
| RP11-473M20.5 | GO:0042304 | 0.00953 | BP |
| RP11-473M20.5 | GO:0045923 | 0.00953 | BP |
| RP11-473M20.5 | GO:0006891 | 0.00953 | BP |
| RP11-473M20.5 | GO:0019317 | 0.00953 | BP |
| RP11-473M20.5 | GO:0042355 | 0.00953 | BP |
| RP11-473M20.5 | GO:0045777 | 0.00953 | BP |
| RP11-473M20.5 | GO:0045940 | 0.00953 | BP |
| RP11-473M20.5 | GO:0046889 | 0.00953 | BP |
| RP11-473M20.5 | GO:0019229 | 0.00984 | BP |
| RP11-473M20.5 | GO:0042354 | 0.00984 | BP |
| RP11-554A11.9 | GO:0002034 | 0.009 | BP |
| RP11-554A11.9 | GO:0002035 | 0.009 | BP |
| RP11-554A11.9 | GO:0003072 | 0.009 | BP |
| RP11-554A11.9 | GO:0003071 | 0.009 | BP |
| RP11-554A11.9 | GO:0003078 | 0.009 | BP |
| RP11-554A11.9 | GO:0003084 | 0.009 | BP |
| RP11-554A11.9 | GO:0001976 | 0.009 | BP |
| RP11-554A11.9 | GO:0001991 | 0.009 | BP |
| RP11-554A11.9 | GO:0002016 | 0.009 | BP |
| RP11-554A11.9 | GO:0034374 | 0.009 | BP |
| RP11-554A11.9 | GO:0042147 | 0.009 | BP |
| RP11-554A11.9 | GO:0044062 | 0.009 | BP |
| RP11-554A11.9 | GO:0003081 | 0.009 | BP |
| RP11-554A11.9 | GO:0009409 | 0.009 | BP |
| RP11-554A11.9 | GO:0045723 | 0.009 | BP |
| RP11-554A11.9 | GO:0009651 | 0.009 | BP |
| RP11-554A11.9 | GO:0010873 | 0.009 | BP |
| RP11-554A11.9 | GO:0030004 | 0.009 | BP |
| RP11-554A11.9 | GO:0003014 | 0.009 | BP |
| RP11-554A11.9 | GO:0010594 | 0.009 | BP |
| RP11-554A11.9 | GO:0010872 | 0.009 | BP |
| RP11-554A11.9 | GO:0001974 | 0.009 | BP |
| RP11-554A11.9 | GO:0006970 | 0.009 | BP |
| RP11-554A11.9 | GO:0042304 | 0.009 | BP |
| RP11-554A11.9 | GO:0045923 | 0.009 | BP |
| RP11-554A11.9 | GO:0006891 | 0.009 | BP |
| RP11-554A11.9 | GO:0019317 | 0.009 | BP |
| RP11-554A11.9 | GO:0042355 | 0.009 | BP |
| RP11-554A11.9 | GO:0045777 | 0.009 | BP |
| RP11-554A11.9 | GO:0045940 | 0.009 | BP |
| RP11-554A11.9 | GO:0046889 | 0.009 | BP |
| RP11-554A11.9 | GO:0019229 | 0.0093 | BP |
| RP11-554A11.9 | GO:0042354 | 0.0093 | BP |
| RP11-650J17.2 | GO:0042147 | 0.00895 | BP |
| RP11-650J17.2 | GO:0006891 | 0.00895 | BP |
| RP11-650J17.2 | GO:0019317 | 0.00895 | BP |
| RP11-650J17.2 | GO:0042355 | 0.00895 | BP |
| RP11-650J17.2 | GO:0006833 | 0.00895 | BP |
| RP11-650J17.2 | GO:0042044 | 0.00895 | BP |
| RP11-650J17.2 | GO:0042354 | 0.00895 | BP |
| RP11-650J17.2 | GO:0006004 | 0.00895 | BP |
| RP11-650J17.2 | GO:0016197 | 0.00895 | BP |
| RP11-650J17.2 | GO:0048839 | 0.00895 | BP |
| RP11-730K11.1 | GO:0019079 | 0.00256 | BP |
| RP11-730K11.1 | GO:0019058 | 0.00256 | BP |
| RP11-730K11.1 | GO:0007163 | 0.00256 | BP |
| RP11-730K11.1 | GO:0022415 | 0.00395 | BP |
| RP11-730K11.1 | GO:0016032 | 0.00395 | BP |
| RP11-730K11.1 | GO:0006935 | 0.0062 | BP |
| RP11-730K11.1 | GO:0042330 | 0.0062 | BP |
| RP11-730K11.1 | GO:0007626 | 0.00659 | BP |
| RP11-730K11.1 | GO:0009611 | 0.00953 | BP |
| RP11-730K11.1 | GO:0006954 | 0.00953 | BP |
| RP11-730K11.1 | GO:0007610 | 0.00953 | BP |
| RP11-77G23.5 | GO:0000768 | 0.00685 | BP |
| RP11-77G23.5 | GO:0006949 | 0.00685 | BP |
| RP11-77G23.5 | GO:0006833 | 0.00685 | BP |
| RP11-77G23.5 | GO:0042044 | 0.00685 | BP |
| RP11-77G23.5 | GO:0007588 | 0.00944 | BP |
| RP11-77M5.1 | GO:0048667 | 0.00874 | BP |
| RP11-77M5.1 | GO:0000904 | 0.00874 | BP |
| RP11-77M5.1 | GO:0007409 | 0.00985 | BP |
| RP3-414A15.2 | GO:0042147 | 0.00756 | BP |
| RP3-414A15.2 | GO:0006891 | 0.00756 | BP |
| RP3-414A15.2 | GO:0019317 | 0.00756 | BP |
| RP3-414A15.2 | GO:0042355 | 0.00756 | BP |
| RP3-414A15.2 | GO:0042354 | 0.00756 | BP |
| RP3-414A15.2 | GO:0006004 | 0.00756 | BP |
| RP3-414A15.2 | GO:0016197 | 0.00756 | BP |
| RP3-414A15.2 | GO:0048839 | 0.00756 | BP |
| RP3-414A15.2 | GO:0016338 | 0.00984 | BP |
| RP3-512E2.2 | GO:0001558 | 0.00969 | BP |
| RP3-512E2.2 | GO:0002034 | 0.00969 | BP |
| RP3-512E2.2 | GO:0002035 | 0.00969 | BP |
| RP3-512E2.2 | GO:0003072 | 0.00969 | BP |
| RP3-512E2.2 | GO:0003071 | 0.00969 | BP |
| RP3-512E2.2 | GO:0003078 | 0.00969 | BP |
| RP3-512E2.2 | GO:0003084 | 0.00969 | BP |
| RP3-512E2.2 | GO:0001976 | 0.00969 | BP |
| RP3-512E2.2 | GO:0001991 | 0.00969 | BP |
| RP3-512E2.2 | GO:0002016 | 0.00969 | BP |
| RP3-512E2.2 | GO:0034374 | 0.00969 | BP |
| RP3-512E2.2 | GO:0042147 | 0.00969 | BP |
| RP3-512E2.2 | GO:0044062 | 0.00969 | BP |
| RP3-512E2.2 | GO:0000768 | 0.00969 | BP |
| RP3-512E2.2 | GO:0003081 | 0.00969 | BP |
| RP3-512E2.2 | GO:0006949 | 0.00969 | BP |
| RP3-512E2.2 | GO:0009409 | 0.00969 | BP |
| RP3-512E2.2 | GO:0045723 | 0.00969 | BP |
| RP3-512E2.2 | GO:0009651 | 0.00969 | BP |
| RP3-512E2.2 | GO:0010873 | 0.00969 | BP |
| RP3-512E2.2 | GO:0030004 | 0.00969 | BP |
| RP3-512E2.2 | GO:0003014 | 0.00969 | BP |
| RP3-512E2.2 | GO:0010594 | 0.00969 | BP |
| RP3-512E2.2 | GO:0010872 | 0.00969 | BP |
| RP3-512E2.2 | GO:0001974 | 0.00969 | BP |
| RP3-512E2.2 | GO:0006970 | 0.00969 | BP |
| RP3-512E2.2 | GO:0042304 | 0.00969 | BP |
| RP3-512E2.2 | GO:0045923 | 0.00969 | BP |
| RP3-512E2.2 | GO:0006891 | 0.00969 | BP |
| RP3-512E2.2 | GO:0019317 | 0.00969 | BP |
| RP3-512E2.2 | GO:0042355 | 0.00969 | BP |
| RP3-512E2.2 | GO:0045777 | 0.00969 | BP |
| RP3-512E2.2 | GO:0045940 | 0.00969 | BP |
| RP3-512E2.2 | GO:0046889 | 0.00969 | BP |
| RP3-512E2.2 | GO:0019229 | 0.00969 | BP |
| RP3-512E2.2 | GO:0042354 | 0.00969 | BP |
| RP3-512E2.2 | GO:0016337 | 0.00969 | BP |
| RP3-512E2.2 | GO:0019218 | 0.00969 | BP |
| RP3-512E2.2 | GO:0034367 | 0.00969 | BP |
| RP3-512E2.2 | GO:0034368 | 0.00969 | BP |
| RP3-512E2.2 | GO:0034369 | 0.00969 | BP |
| RP3-512E2.2 | GO:0048771 | 0.00969 | BP |
| RP3-512E2.2 | GO:0043065 | 0.00969 | BP |
| RP3-512E2.2 | GO:0043068 | 0.00969 | BP |
| RP3-512E2.2 | GO:0010942 | 0.00969 | BP |
| RP3-512E2.2 | GO:0003044 | 0.00969 | BP |
| RP3-512E2.2 | GO:0010743 | 0.00969 | BP |
| RP3-512E2.2 | GO:0042310 | 0.00969 | BP |
| RP3-522J7.6 | GO:0007588 | 0.00476 | BP |
| RP3-522J7.6 | GO:0002034 | 0.00944 | BP |
| RP3-522J7.6 | GO:0002035 | 0.00944 | BP |
| RP3-522J7.6 | GO:0003072 | 0.00944 | BP |
| RP3-522J7.6 | GO:0046903 | 0.00944 | BP |
| RP3-522J7.6 | GO:0003071 | 0.00944 | BP |
| RP3-522J7.6 | GO:0003078 | 0.00944 | BP |
| RP3-522J7.6 | GO:0003084 | 0.00944 | BP |
| RP3-522J7.6 | GO:0001976 | 0.00944 | BP |
| RP3-522J7.6 | GO:0001991 | 0.00944 | BP |
| RP3-522J7.6 | GO:0002016 | 0.00944 | BP |
| RP3-522J7.6 | GO:0034374 | 0.00944 | BP |
| RP3-522J7.6 | GO:0042147 | 0.00944 | BP |
| RP3-522J7.6 | GO:0044062 | 0.00944 | BP |
| RP3-522J7.6 | GO:0003081 | 0.00944 | BP |
| RP3-522J7.6 | GO:0009409 | 0.00944 | BP |
| RP3-522J7.6 | GO:0045723 | 0.00944 | BP |
| RP3-522J7.6 | GO:0009651 | 0.00944 | BP |
| RP3-522J7.6 | GO:0010873 | 0.00944 | BP |
| RP3-522J7.6 | GO:0030004 | 0.00944 | BP |
| RP3-522J7.6 | GO:0003014 | 0.00944 | BP |
| RP3-522J7.6 | GO:0010594 | 0.00944 | BP |
| RP3-522J7.6 | GO:0010872 | 0.00944 | BP |
| RP3-522J7.6 | GO:0001974 | 0.00944 | BP |
| RP3-522J7.6 | GO:0006970 | 0.00944 | BP |
| RP3-522J7.6 | GO:0042304 | 0.00944 | BP |
| RP3-522J7.6 | GO:0045923 | 0.00944 | BP |
| RP3-522J7.6 | GO:0006891 | 0.00944 | BP |
| RP3-522J7.6 | GO:0019317 | 0.00944 | BP |
| RP3-522J7.6 | GO:0042355 | 0.00944 | BP |
| RP3-522J7.6 | GO:0045777 | 0.00944 | BP |
| RP3-522J7.6 | GO:0045940 | 0.00944 | BP |
| RP3-522J7.6 | GO:0046889 | 0.00944 | BP |
| RP4-778K6.3 | GO:0002034 | 0.00969 | BP |
| RP4-778K6.3 | GO:0002035 | 0.00969 | BP |
| RP4-778K6.3 | GO:0003072 | 0.00969 | BP |
| RP4-778K6.3 | GO:0003071 | 0.00969 | BP |
| RP4-778K6.3 | GO:0003078 | 0.00969 | BP |
| RP4-778K6.3 | GO:0003084 | 0.00969 | BP |
| RP4-778K6.3 | GO:0001976 | 0.00969 | BP |
| RP4-778K6.3 | GO:0001991 | 0.00969 | BP |
| RP4-778K6.3 | GO:0002016 | 0.00969 | BP |
| RP4-778K6.3 | GO:0034374 | 0.00969 | BP |
| RP4-778K6.3 | GO:0042147 | 0.00969 | BP |
| RP4-778K6.3 | GO:0044062 | 0.00969 | BP |
| RP4-778K6.3 | GO:0003081 | 0.00969 | BP |
| RP4-778K6.3 | GO:0009409 | 0.00969 | BP |
| RP4-778K6.3 | GO:0045723 | 0.00969 | BP |
| RP4-778K6.3 | GO:0009651 | 0.00969 | BP |
| RP4-778K6.3 | GO:0010873 | 0.00969 | BP |
| RP4-778K6.3 | GO:0030004 | 0.00969 | BP |
| RP4-778K6.3 | GO:0003014 | 0.00969 | BP |
| RP4-778K6.3 | GO:0010594 | 0.00969 | BP |
| RP4-778K6.3 | GO:0010872 | 0.00969 | BP |
| RP4-778K6.3 | GO:0001974 | 0.00969 | BP |
| RP4-778K6.3 | GO:0006970 | 0.00969 | BP |
| RP4-778K6.3 | GO:0042304 | 0.00969 | BP |
| RP4-778K6.3 | GO:0045923 | 0.00969 | BP |
| RP4-778K6.3 | GO:0006891 | 0.00969 | BP |
| RP4-778K6.3 | GO:0019317 | 0.00969 | BP |
| RP4-778K6.3 | GO:0042355 | 0.00969 | BP |
| RP4-778K6.3 | GO:0045777 | 0.00969 | BP |
| RP4-778K6.3 | GO:0045940 | 0.00969 | BP |
| RP4-778K6.3 | GO:0046889 | 0.00969 | BP |
| RP4-778K6.3 | GO:0019229 | 0.00969 | BP |
| RP4-778K6.3 | GO:0042354 | 0.00969 | BP |
| RP4-778K6.3 | GO:0019218 | 0.00969 | BP |
| RP4-778K6.3 | GO:0034367 | 0.00969 | BP |
| RP4-778K6.3 | GO:0034368 | 0.00969 | BP |
| RP4-778K6.3 | GO:0034369 | 0.00969 | BP |
| RP4-778K6.3 | GO:0048771 | 0.00969 | BP |
| RP4-778K6.3 | GO:0003044 | 0.00969 | BP |
| RP4-778K6.3 | GO:0010743 | 0.00969 | BP |
| RP4-778K6.3 | GO:0042310 | 0.00969 | BP |
| RP4-778K6.3 | GO:0001990 | 0.00969 | BP |
| RP4-778K6.3 | GO:0006004 | 0.00969 | BP |
| RP4-778K6.3 | GO:0016197 | 0.00969 | BP |
| RP4-778K6.3 | GO:0019217 | 0.00969 | BP |
| RP4-778K6.3 | GO:0048839 | 0.00969 | BP |
| RP4-778K6.3 | GO:0050727 | 0.00969 | BP |
| RP4-778K6.3 | GO:0050886 | 0.00969 | BP |
| SRGAP3-AS4 | GO:0006833 | 0.00384 | BP |
| SRGAP3-AS4 | GO:0042044 | 0.00384 | BP |
| SRGAP3-AS4 | GO:0007588 | 0.00442 | BP |
| SRGAP3-AS4 | GO:0048545 | 0.00886 | BP |
| SRGAP3-AS4 | GO:0046903 | 0.00886 | BP |
| SRGAP3-AS4 | GO:0007601 | 0.00886 | BP |
| SRGAP3-AS4 | GO:0050953 | 0.00886 | BP |
| UBOX5-AS1 | GO:0030029 | 0.00996 | BP |
| UBOX5-AS1 | GO:0030036 | 0.00996 | BP |
| UBOX5-AS1 | GO:0000902 | 0.00996 | BP |
| ZBTB20-AS3 | GO:0042147 | 0.00504 | BP |
| ZBTB20-AS3 | GO:0006891 | 0.00504 | BP |
| ZBTB20-AS3 | GO:0019317 | 0.00504 | BP |
| ZBTB20-AS3 | GO:0042355 | 0.00504 | BP |
| ZBTB20-AS3 | GO:0042354 | 0.00504 | BP |
| ZBTB20-AS3 | GO:0006004 | 0.00504 | BP |
| ZBTB20-AS3 | GO:0016197 | 0.00504 | BP |
| ZBTB20-AS3 | GO:0048839 | 0.00504 | BP |
| ZBTB20-AS3 | GO:0016338 | 0.00657 | BP |
| AC015977.6 | GO:0016324 | 0.0097 | CC |
| AC064875.2 | GO:0001673 | 0.000299 | CC |
| AC064875.2 | GO:0043073 | 0.000299 | CC |
| APOA1-AS | GO:0016327 | 0.00109 | CC |
| APOA1-AS | GO:0043218 | 0.00209 | CC |
| CCDC13-AS1 | GO:0043218 | 0.0012 | CC |
| CTA-929C8.8 | GO:0016327 | 0.0014 | CC |
| CTA-929C8.8 | GO:0043218 | 0.00292 | CC |
| CTA-929C8.8 | GO:0031201 | 0.00969 | CC |
| CTC-506B8.1 | GO:0030054 | 0.0012 | CC |
| CTD-2270P14.5 | GO:0043218 | 0.00449 | CC |
| CTD-2270P14.5 | GO:0030027 | 0.00797 | CC |
| CTD-2270P14.5 | GO:0042383 | 0.00797 | CC |
| CTD-2270P14.5 | GO:0031252 | 0.00933 | CC |
| CTD-2270P14.5 | GO:0031253 | 0.00984 | CC |
| FUT8-AS1 | GO:0001673 | 0.00419 | CC |
| FUT8-AS1 | GO:0043073 | 0.00419 | CC |
| GNG12-AS1 | GO:0016327 | 0.00299 | CC |
| GNG12-AS1 | GO:0043218 | 0.00344 | CC |
| GS1-124K5.3 | GO:0005788 | 0.00523 | CC |
| LINC01266 | GO:0001673 | 0.00538 | CC |
| LINC01266 | GO:0043073 | 0.00538 | CC |
| LINC01266 | GO:0031201 | 0.00895 | CC |
| LINC01533 | GO:0001673 | 0.000599 | CC |
| LINC01533 | GO:0043073 | 0.000599 | CC |
| MIR3180-2 | GO:0001673 | 0.00568 | CC |
| MIR3180-2 | GO:0043073 | 0.00568 | CC |
| MIR3180-3 | GO:0001673 | 0.00568 | CC |
| MIR3180-3 | GO:0043073 | 0.00568 | CC |
| RAB30-AS1 | GO:0016327 | 0.00109 | CC |
| RAB30-AS1 | GO:0043218 | 0.00209 | CC |
| RP11-120D5.1 | GO:0016327 | 0.000677 | CC |
| RP11-120D5.1 | GO:0043218 | 0.002395 | CC |
| RP11-120D5.1 | GO:0005783 | 0.006371 | CC |
| RP11-120D5.1 | GO:0042383 | 0.006371 | CC |
| RP11-181C3.1 | GO:0001673 | 0.000599 | CC |
| RP11-181C3.1 | GO:0043073 | 0.000599 | CC |
| RP11-264K23.1 | GO:0001673 | 0.00568 | CC |
| RP11-264K23.1 | GO:0043073 | 0.00568 | CC |
| RP11-283G6.5 | GO:0001673 | 0.00508 | CC |
| RP11-283G6.5 | GO:0043073 | 0.00508 | CC |
| RP11-342D11.2 | GO:0043218 | 0.00314 | CC |
| RP11-342D11.2 | GO:0042383 | 0.00837 | CC |
| RP11-342D11.2 | GO:0031091 | 0.00905 | CC |
| RP11-388K2.1 | GO:0005923 | 0.00598 | CC |
| RP11-388K2.1 | GO:0070160 | 0.00598 | CC |
| RP11-388K2.1 | GO:0043296 | 0.00598 | CC |
| RP11-388K2.1 | GO:0016327 | 0.00747 | CC |
| RP11-420K8.1 | GO:0001673 | 0.0015 | CC |
| RP11-420K8.1 | GO:0043073 | 0.0015 | CC |
| RP11-453A12.1 | GO:0001673 | 0.0018 | CC |
| RP11-453A12.1 | GO:0043073 | 0.0018 | CC |
| RP11-473M20.5 | GO:0016327 | 0.00182 | CC |
| RP11-473M20.5 | GO:0043218 | 0.00269 | CC |
| RP11-554A11.9 | GO:0016327 | 0.00162 | CC |
| RP11-554A11.9 | GO:0043218 | 0.00254 | CC |
| RP11-650J17.2 | GO:0030054 | 0.00895 | CC |
| RP11-650J17.2 | GO:0031201 | 0.00895 | CC |
| RP11-77G23.5 | GO:0042995 | 0.00724 | CC |
| RP11-77G23.5 | GO:0030054 | 0.00724 | CC |
| RP11-77G23.5 | GO:0030027 | 0.00746 | CC |
| RP11-77M5.1 | GO:0001673 | 0.000898 | CC |
| RP11-77M5.1 | GO:0043073 | 0.000898 | CC |
| RP3-414A15.2 | GO:0031201 | 0.00672 | CC |
| RP3-512E2.2 | GO:0044421 | 0.00929 | CC |
| RP3-522J7.6 | GO:0016327 | 0.00203 | CC |
| RP3-522J7.6 | GO:0043218 | 0.00284 | CC |
| RP4-778K6.3 | GO:0031201 | 0.00969 | CC |
| SRGAP3-AS4 | GO:0005788 | 0.00737 | CC |
| SRGAP3-AS4 | GO:0016324 | 0.00737 | CC |
| SRGAP3-AS4 | GO:0016323 | 0.00737 | CC |
| SRGAP3-AS4 | GO:0045177 | 0.00737 | CC |
| UBOX5-AS1 | GO:0030027 | 0.00329 | CC |
| UBOX5-AS1 | GO:0031252 | 0.00329 | CC |
| UBOX5-AS1 | GO:0031253 | 0.00329 | CC |
| ZBTB20-AS3 | GO:0031201 | 0.00895 | CC |
| ZBTB20-AS3 | GO:0005923 | 0.00895 | CC |
| ZBTB20-AS3 | GO:0070160 | 0.00895 | CC |
| ZBTB20-AS3 | GO:0043296 | 0.00895 | CC |
| AC015977.6 | GO:0048154 | 0.0015 | MF |
| AC015977.6 | GO:0005372 | 0.0015 | MF |
| AC015977.6 | GO:0015250 | 0.0015 | MF |
| AC015977.6 | GO:0048306 | 0.0015 | MF |
| APOA1-AS | GO:0004745 | 0.00766 | MF |
| APOA1-AS | GO:0046920 | 0.00766 | MF |
| APOA1-AS | GO:0008417 | 0.00766 | MF |
| CCDC13-AS1 | GO:0048154 | 0.00478 | MF |
| CCDC13-AS1 | GO:0004745 | 0.00478 | MF |
| CCDC13-AS1 | GO:0005372 | 0.00478 | MF |
| CCDC13-AS1 | GO:0015250 | 0.00478 | MF |
| CCDC13-AS1 | GO:0048306 | 0.00478 | MF |
| CCDC13-AS1 | GO:0060090 | 0.00875 | MF |
| CTA-929C8.8 | GO:0048154 | 0.00712 | MF |
| CTA-929C8.8 | GO:0046920 | 0.00712 | MF |
| CTA-929C8.8 | GO:0048306 | 0.00712 | MF |
| CTA-929C8.8 | GO:0008417 | 0.00712 | MF |
| CTC-506B8.1 | GO:0048154 | 0.00994 | MF |
| CTC-506B8.1 | GO:0004745 | 0.00994 | MF |
| CTC-506B8.1 | GO:0046920 | 0.00994 | MF |
| CTC-506B8.1 | GO:0005372 | 0.00994 | MF |
| CTC-506B8.1 | GO:0015250 | 0.00994 | MF |
| CTC-506B8.1 | GO:0048306 | 0.00994 | MF |
| CTD-2207A17.1 | GO:0001871 | 0.00561 | MF |
| CTD-2207A17.1 | GO:0030247 | 0.00561 | MF |
| CTD-2207A17.1 | GO:0005539 | 0.00613 | MF |
| CTD-2270P14.5 | GO:0048154 | 0.00539 | MF |
| CTD-2270P14.5 | GO:0004745 | 0.00539 | MF |
| CTD-2270P14.5 | GO:0048306 | 0.00598 | MF |
| CTD-2270P14.5 | GO:0004089 | 0.00673 | MF |
| CTD-2270P14.5 | GO:0060090 | 0.00776 | MF |
| CTD-2270P14.5 | GO:0016836 | 0.00776 | MF |
| FAM225A | GO:0048154 | 0.00349 | MF |
| FAM225A | GO:0004745 | 0.00349 | MF |
| FAM225A | GO:0005372 | 0.00349 | MF |
| FAM225A | GO:0015250 | 0.00349 | MF |
| FAM225A | GO:0048306 | 0.00349 | MF |
| FUT8-AS1 | GO:0046920 | 0.00835 | MF |
| FUT8-AS1 | GO:0008417 | 0.00835 | MF |
| FUT8-AS1 | GO:0005149 | 0.00835 | MF |
| GNG12-AS1 | GO:0048154 | 0.00915 | MF |
| GNG12-AS1 | GO:0004745 | 0.00915 | MF |
| GNG12-AS1 | GO:0046920 | 0.00915 | MF |
| GS1-124K5.3 | GO:0004745 | 0.00359 | MF |
| GS1-124K5.3 | GO:0001871 | 0.00498 | MF |
| GS1-124K5.3 | GO:0030247 | 0.00498 | MF |
| GS1-124K5.3 | GO:0005539 | 0.00613 | MF |
| LINC00907 | GO:0003707 | 0.00718 | MF |
| LINC00907 | GO:0004879 | 0.00718 | MF |
| LINC00907 | GO:0003690 | 0.00737 | MF |
| LINC00907 | GO:0043566 | 0.00791 | MF |
| LINC01266 | GO:0046920 | 0.00657 | MF |
| LINC01266 | GO:0008417 | 0.00657 | MF |
| LINC01580 | GO:0046920 | 0.00766 | MF |
| LINC01580 | GO:0008417 | 0.00766 | MF |
| MCM8-AS1 | GO:0005149 | 0.00359 | MF |
| MCM8-AS1 | GO:0008009 | 0.00439 | MF |
| MCM8-AS1 | GO:0042379 | 0.00439 | MF |
| MIR3180-2 | GO:0048154 | 0.00944 | MF |
| MIR3180-2 | GO:0046920 | 0.00944 | MF |
| MIR3180-2 | GO:0048306 | 0.00944 | MF |
| MIR3180-3 | GO:0048154 | 0.00944 | MF |
| MIR3180-3 | GO:0046920 | 0.00944 | MF |
| MIR3180-3 | GO:0048306 | 0.00944 | MF |
| RAB30-AS1 | GO:0048154 | 0.00766 | MF |
| RAB30-AS1 | GO:0046920 | 0.00766 | MF |
| RAB30-AS1 | GO:0048306 | 0.00766 | MF |
| RAB30-AS1 | GO:0008417 | 0.00766 | MF |
| RP1-122O8.7 | GO:0046920 | 0.0012 | MF |
| RP1-122O8.7 | GO:0008417 | 0.0012 | MF |
| RP1-122O8.7 | GO:0005149 | 0.0012 | MF |
| RP11-120D5.1 | GO:0046920 | 0.00597 | MF |
| RP11-120D5.1 | GO:0008417 | 0.00597 | MF |
| RP11-120D5.1 | GO:0004089 | 0.00597 | MF |
| RP11-130C19.3 | GO:0004745 | 0.00498 | MF |
| RP11-130C19.3 | GO:0005372 | 0.00498 | MF |
| RP11-130C19.3 | GO:0015250 | 0.00498 | MF |
| RP11-130C19.3 | GO:0001871 | 0.00746 | MF |
| RP11-130C19.3 | GO:0030247 | 0.00746 | MF |
| RP11-264K23.1 | GO:0048154 | 0.00944 | MF |
| RP11-264K23.1 | GO:0046920 | 0.00944 | MF |
| RP11-264K23.1 | GO:0005372 | 0.00944 | MF |
| RP11-264K23.1 | GO:0015250 | 0.00944 | MF |
| RP11-264K23.1 | GO:0048306 | 0.00944 | MF |
| RP11-268P4.4 | GO:0005372 | 0.00249 | MF |
| RP11-268P4.4 | GO:0015250 | 0.00249 | MF |
| RP11-283G6.5 | GO:0048154 | 0.0093 | MF |
| RP11-283G6.5 | GO:0046920 | 0.0093 | MF |
| RP11-283G6.5 | GO:0048306 | 0.0093 | MF |
| RP11-283G6.5 | GO:0008417 | 0.0093 | MF |
| RP11-342D11.2 | GO:0048154 | 0.00611 | MF |
| RP11-342D11.2 | GO:0005372 | 0.00611 | MF |
| RP11-342D11.2 | GO:0015250 | 0.00611 | MF |
| RP11-342D11.2 | GO:0048306 | 0.00611 | MF |
| RP11-342D11.2 | GO:0004089 | 0.00732 | MF |
| RP11-342D11.2 | GO:0060090 | 0.00894 | MF |
| RP11-342D11.2 | GO:0016836 | 0.00905 | MF |
| RP11-388K2.1 | GO:0046920 | 0.00165 | MF |
| RP11-388K2.1 | GO:0008417 | 0.00165 | MF |
| RP11-420K8.1 | GO:0046920 | 0.00299 | MF |
| RP11-420K8.1 | GO:0008417 | 0.00299 | MF |
| RP11-420K8.1 | GO:0005149 | 0.00299 | MF |
| RP11-453A12.1 | GO:0048154 | 0.00599 | MF |
| RP11-453A12.1 | GO:0048306 | 0.00717 | MF |
| RP11-453A12.1 | GO:0004623 | 0.00717 | MF |
| RP11-453A12.1 | GO:0003707 | 0.00717 | MF |
| RP11-453A12.1 | GO:0004879 | 0.00717 | MF |
| RP11-473M20.5 | GO:0048154 | 0.00984 | MF |
| RP11-473M20.5 | GO:0046920 | 0.00984 | MF |
| RP11-473M20.5 | GO:0048306 | 0.00984 | MF |
| RP11-473M20.5 | GO:0008417 | 0.00984 | MF |
| RP11-554A11.9 | GO:0048154 | 0.0093 | MF |
| RP11-554A11.9 | GO:0046920 | 0.0093 | MF |
| RP11-554A11.9 | GO:0048306 | 0.0093 | MF |
| RP11-554A11.9 | GO:0008417 | 0.0093 | MF |
| RP11-650J17.2 | GO:0046920 | 0.00657 | MF |
| RP11-650J17.2 | GO:0005372 | 0.00657 | MF |
| RP11-650J17.2 | GO:0015250 | 0.00657 | MF |
| RP11-650J17.2 | GO:0008417 | 0.00657 | MF |
| RP11-730K11.1 | GO:0008009 | 0.00439 | MF |
| RP11-730K11.1 | GO:0042379 | 0.00439 | MF |
| RP11-730K11.1 | GO:0005125 | 0.00865 | MF |
| RP11-730K11.1 | GO:0004672 | 0.00993 | MF |
| RP11-77G23.5 | GO:0004745 | 0.00498 | MF |
| RP11-77G23.5 | GO:0005372 | 0.00498 | MF |
| RP11-77G23.5 | GO:0015250 | 0.00498 | MF |
| RP11-77M5.1 | GO:0048154 | 0.00359 | MF |
| RP11-77M5.1 | GO:0048306 | 0.00449 | MF |
| RP11-77M5.1 | GO:0004623 | 0.00688 | MF |
| RP11-77M5.1 | GO:0004620 | 0.0097 | MF |
| RP11-77M5.1 | GO:0016298 | 0.0097 | MF |
| RP11-77M5.1 | GO:0004091 | 0.0097 | MF |
| RP3-414A15.2 | GO:0046920 | 0.00493 | MF |
| RP3-414A15.2 | GO:0008417 | 0.00493 | MF |
| RP3-512E2.2 | GO:0046920 | 0.00895 | MF |
| RP3-512E2.2 | GO:0008417 | 0.00895 | MF |
| RP3-512E2.2 | GO:0005149 | 0.00895 | MF |
| RP3-522J7.6 | GO:0048154 | 0.00944 | MF |
| RP3-522J7.6 | GO:0046920 | 0.00944 | MF |
| RP3-522J7.6 | GO:0005372 | 0.00944 | MF |
| RP3-522J7.6 | GO:0015250 | 0.00944 | MF |
| RP3-522J7.6 | GO:0048306 | 0.00944 | MF |
| RP4-778K6.3 | GO:0046920 | 0.00776 | MF |
| RP4-778K6.3 | GO:0008417 | 0.00776 | MF |
| RP4-778K6.3 | GO:0005149 | 0.00776 | MF |
| RP5-943J3.2 | GO:0004623 | 0.00648 | MF |
| RP5-943J3.2 | GO:0004620 | 0.00648 | MF |
| RP5-943J3.2 | GO:0016298 | 0.00648 | MF |
| RP5-943J3.2 | GO:0004091 | 0.00648 | MF |
| SRGAP3-AS4 | GO:0004745 | 0.000998 | MF |
| SRGAP3-AS4 | GO:0005372 | 0.000998 | MF |
| SRGAP3-AS4 | GO:0015250 | 0.000998 | MF |
| UBOX5-AS1 | GO:0004866 | 0.00807 | MF |
| UBOX5-AS1 | GO:0030414 | 0.00807 | MF |
| ZBTB20-AS3 | GO:0046920 | 0.00329 | MF |
| ZBTB20-AS3 | GO:0008417 | 0.00329 | MF |
| CCDC13-AS1 | The Visual Cycle | 0.00597 | pathway |
| CTD-2207A17.1 | Chondroitin Sulfate Degradation (Metazoa) | 0.00292 | pathway |
| CTD-2207A17.1 | Dermatan Sulfate Degradation (Metazoa) | 0.00292 | pathway |
| CTD-2207A17.1 | Glioma Invasiveness Signaling | 0.00851 | pathway |
| CTD-2270P14.5 | The Visual Cycle | 0.00865 | pathway |
| CTD-2270P14.5 | Retinoate Biosynthesis I | 0.00865 | pathway |
| FAM225A | The Visual Cycle | 0.00523 | pathway |
| GS1-124K5.3 | Chondroitin Sulfate Degradation (Metazoa) | 0.00374 | pathway |
| GS1-124K5.3 | Dermatan Sulfate Degradation (Metazoa) | 0.00374 | pathway |
| GS1-124K5.3 | The Visual Cycle | 0.00374 | pathway |
| GS1-124K5.3 | Retinoate Biosynthesis I | 0.00542 | pathway |
| GS1-124K5.3 | Glioma Invasiveness Signaling | 0.00851 | pathway |
| LINC00907 | Role of Oct4 in Mammalian Embryonic Stem Cell Pluripotency | 0.00642 | pathway |
| MCM8-AS1 | iNOS Signaling | 0.00822 | pathway |
| MCM8-AS1 | Toll-like Receptor Signaling | 0.00822 | pathway |
| MCM8-AS1 | IL-1 Signaling | 0.00906 | pathway |
| MIR3180-2 | p38 MAPK Signaling | 0.00455 | pathway |
| MIR3180-3 | p38 MAPK Signaling | 0.00455 | pathway |
| RP1-122O8.7 | iNOS Signaling | 0.00822 | pathway |
| RP1-122O8.7 | Toll-like Receptor Signaling | 0.00822 | pathway |
| RP1-122O8.7 | IL-1 Signaling | 0.00906 | pathway |
| RP11-120D5.1 | Cell Cycle Control of Chromosomal Replication | 0.00835 | pathway |
| RP11-130C19.3 | Chondroitin Sulfate Degradation (Metazoa) | 0.00598 | pathway |
| RP11-130C19.3 | Dermatan Sulfate Degradation (Metazoa) | 0.00598 | pathway |
| RP11-130C19.3 | The Visual Cycle | 0.00598 | pathway |
| RP11-130C19.3 | Retinoate Biosynthesis I | 0.00865 | pathway |
| RP11-342D11.2 | Cell Cycle Control of Chromosomal Replication | 0.00731 | pathway |
| RP11-453A12.1 | MIF-mediated Glucocorticoid Regulation | 0.00954 | pathway |
| RP11-77G23.5 | Chondroitin Sulfate Degradation (Metazoa) | 0.00746 | pathway |
| RP11-77G23.5 | Dermatan Sulfate Degradation (Metazoa) | 0.00746 | pathway |
| RP11-77G23.5 | The Visual Cycle | 0.00746 | pathway |
| RP11-77M5.1 | MIF-mediated Glucocorticoid Regulation | 0.0097 | pathway |
| RP11-77M5.1 | MIF Regulation of Innate Immunity | 0.0097 | pathway |
| RP11-77M5.1 | Phospholipases | 0.0097 | pathway |
| RP11-77M5.1 | Eicosanoid Signaling | 0.0097 | pathway |
| RP11-77M5.1 | Role of MAPK Signaling in the Pathogenesis of Influenza | 0.0097 | pathway |
| RP3-512E2.2 | Leukocyte Extravasation Signaling | 0.00969 | pathway |
| RP3-512E2.2 | Chondroitin Sulfate Degradation (Metazoa) | 0.00969 | pathway |
| RP3-512E2.2 | Dermatan Sulfate Degradation (Metazoa) | 0.00969 | pathway |
| RP5-943J3.2 | MIF-mediated Glucocorticoid Regulation | 0.00872 | pathway |
| RP5-943J3.2 | MIF Regulation of Innate Immunity | 0.00872 | pathway |
| RP5-943J3.2 | Phospholipases | 0.00872 | pathway |
| RP5-943J3.2 | Eicosanoid Signaling | 0.00872 | pathway |
| RP5-943J3.2 | Role of MAPK Signaling in the Pathogenesis of Influenza | 0.00872 | pathway |
| RP5-943J3.2 | VEGF Family Ligand-Receptor Interactions | 0.00872 | pathway |
| RP5-943J3.2 | Antioxidant Action of Vitamin C | 0.00916 | pathway |
| SRGAP3-AS4 | The Visual Cycle | 0.00289 | pathway |
| SRGAP3-AS4 | Retinoate Biosynthesis I | 0.00289 | pathway |

**Table S6** Top 4 ranked candidate miRNAs using miRanda algorithm.

| rank | Var1 | Freq |
| --- | --- | --- |
| 1 | hsa-miR-638 | 35 |
| 1 | hsa-miR-6724 | 35 |
| 1 | hsa-miR-6752 | 35 |
| 1 | hsa-miR-6775 | 35 |
| 2 | hsa-miR-10392 | 34 |
| 2 | hsa-miR-1229 | 34 |
| 2 | hsa-miR-197 | 34 |
| 2 | hsa-miR-3147 | 34 |
| 2 | hsa-miR-4507 | 34 |
| 2 | hsa-miR-4649 | 34 |
| 2 | hsa-miR-4651 | 34 |
| 2 | hsa-miR-4656 | 34 |
| 2 | hsa-miR-4722 | 34 |
| 2 | hsa-miR-4763 | 34 |
| 2 | hsa-miR-6089 | 34 |
| 2 | hsa-miR-6751 | 34 |
| 2 | hsa-miR-6756 | 34 |
| 2 | hsa-miR-6765 | 34 |
| 2 | hsa-miR-6787 | 34 |
| 2 | hsa-miR-6796 | 34 |
| 2 | hsa-miR-6798 | 34 |
| 2 | hsa-miR-6803 | 34 |
| 2 | hsa-miR-6848 | 34 |
| 2 | hsa-miR-8089 | 34 |
| 2 | hsa-miR-939 | 34 |
| 3 | hsa-miR-1207 | 33 |
| 3 | hsa-miR-1226 | 33 |
| 3 | hsa-miR-1228 | 33 |
| 3 | hsa-miR-1343 | 33 |
| 3 | hsa-miR-149 | 33 |
| 3 | hsa-miR-2861 | 33 |
| 3 | hsa-miR-3137 | 33 |
| 3 | hsa-miR-328 | 33 |
| 3 | hsa-miR-3620 | 33 |
| 3 | hsa-miR-3663 | 33 |
| 3 | hsa-miR-3677 | 33 |
| 3 | hsa-miR-3940 | 33 |
| 3 | hsa-miR-3960 | 33 |
| 3 | hsa-miR-4632 | 33 |
| 3 | hsa-miR-4640 | 33 |
| 3 | hsa-miR-4685 | 33 |
| 3 | hsa-miR-4687 | 33 |
| 3 | hsa-miR-4706 | 33 |
| 3 | hsa-miR-4726 | 33 |
| 3 | hsa-miR-4734 | 33 |
| 3 | hsa-miR-4741 | 33 |
| 3 | hsa-miR-4743 | 33 |
| 3 | hsa-miR-5001 | 33 |
| 3 | hsa-miR-608 | 33 |
| 3 | hsa-miR-612 | 33 |
| 3 | hsa-miR-637 | 33 |
| 3 | hsa-miR-658 | 33 |
| 3 | hsa-miR-6722 | 33 |
| 3 | hsa-miR-6726 | 33 |
| 3 | hsa-miR-6727 | 33 |
| 3 | hsa-miR-6729 | 33 |
| 3 | hsa-miR-6746 | 33 |
| 3 | hsa-miR-6749 | 33 |
| 3 | hsa-miR-6777 | 33 |
| 3 | hsa-miR-6782 | 33 |
| 3 | hsa-miR-6786 | 33 |
| 3 | hsa-miR-6789 | 33 |
| 3 | hsa-miR-6793 | 33 |
| 3 | hsa-miR-6795 | 33 |
| 3 | hsa-miR-6813 | 33 |
| 3 | hsa-miR-6816 | 33 |
| 3 | hsa-miR-6819 | 33 |
| 3 | hsa-miR-6855 | 33 |
| 3 | hsa-miR-6871 | 33 |
| 3 | hsa-miR-762 | 33 |
| 3 | hsa-miR-8069 | 33 |
| 4 | hsa-miR-10394 | 32 |
| 4 | hsa-miR-1183 | 32 |
| 4 | hsa-miR-12115 | 32 |
| 4 | hsa-miR-12119 | 32 |
| 4 | hsa-miR-1225 | 32 |
| 4 | hsa-miR-1233 | 32 |
| 4 | hsa-miR-1292 | 32 |
| 4 | hsa-miR-1538 | 32 |
| 4 | hsa-miR-1587 | 32 |
| 4 | hsa-miR-2277 | 32 |
| 4 | hsa-miR-3085 | 32 |
| 4 | hsa-miR-3180 | 32 |
| 4 | hsa-miR-3187 | 32 |
| 4 | hsa-miR-3621 | 32 |
| 4 | hsa-miR-3944 | 32 |
| 4 | hsa-miR-4498 | 32 |
| 4 | hsa-miR-4695 | 32 |
| 4 | hsa-miR-4707 | 32 |
| 4 | hsa-miR-4745 | 32 |
| 4 | hsa-miR-4758 | 32 |
| 4 | hsa-miR-4783 | 32 |
| 4 | hsa-miR-4787 | 32 |
| 4 | hsa-miR-5088 | 32 |
| 4 | hsa-miR-5189 | 32 |
| 4 | hsa-miR-5787 | 32 |
| 4 | hsa-miR-6511a | 32 |
| 4 | hsa-miR-6737 | 32 |
| 4 | hsa-miR-6741 | 32 |
| 4 | hsa-miR-6753 | 32 |
| 4 | hsa-miR-6762 | 32 |
| 4 | hsa-miR-6771 | 32 |
| 4 | hsa-miR-6791 | 32 |
| 4 | hsa-miR-6794 | 32 |
| 4 | hsa-miR-6810 | 32 |
| 4 | hsa-miR-6812 | 32 |
| 4 | hsa-miR-6821 | 32 |
| 4 | hsa-miR-6851 | 32 |
| 4 | hsa-miR-6858 | 32 |
| 4 | hsa-miR-6889 | 32 |
| 4 | hsa-miR-6893 | 32 |
| 4 | hsa-miR-6894 | 32 |
| 4 | hsa-miR-7110 | 32 |
| 4 | hsa-miR-7160 | 32 |
| 4 | hsa-miR-769 | 32 |
| 4 | hsa-miR-8075 | 32 |

**Table S7** Top 4 ranked candidate miRNAs by searching the starBase database.

| rank | miRNA | Freq |
| --- | --- | --- |
| 1 | hsa-miR-520d | 11 |
| 1 | hsa-miR-582 | 11 |
| 1 | hsa-miR-576 | 11 |
| 2 | hsa-miR-519b | 10 |
| 2 | hsa-miR-519c | 10 |
| 2 | hsa-miR-520c | 10 |
| 2 | hsa-miR-545 | 10 |
| 2 | hsa-miR-199a | 10 |
| 2 | hsa-miR-199b | 10 |
| 2 | hsa-miR-129 | 10 |
| 2 | hsa-miR-450b | 10 |
| 2 | hsa-miR-495 | 10 |
| 2 | hsa-miR-524 | 10 |
| 2 | hsa-miR-579 | 10 |
| 2 | hsa-miR-641 | 10 |
| 3 | hsa-miR-330 | 9 |
| 3 | hsa-miR-520a | 9 |
| 3 | hsa-miR-224 | 9 |
| 3 | hsa-miR-212 | 9 |
| 3 | hsa-miR-214 | 9 |
| 3 | hsa-miR-485 | 9 |
| 3 | hsa-miR-526b | 9 |
| 3 | hsa-miR-106a | 9 |
| 3 | hsa-miR-106b | 9 |
| 3 | hsa-miR-17 | 9 |
| 3 | hsa-miR-181a | 9 |
| 3 | hsa-miR-181b | 9 |
| 3 | hsa-miR-181c | 9 |
| 3 | hsa-miR-181d | 9 |
| 3 | hsa-miR-186 | 9 |
| 3 | hsa-miR-20a | 9 |
| 3 | hsa-miR-20b | 9 |
| 3 | hsa-miR-23a | 9 |
| 3 | hsa-miR-23b | 9 |
| 3 | hsa-miR-331 | 9 |
| 3 | hsa-miR-340 | 9 |
| 3 | hsa-miR-433 | 9 |
| 3 | hsa-miR-506 | 9 |
| 3 | hsa-miR-519d | 9 |
| 3 | hsa-miR-93 | 9 |
| 4 | hsa-miR-9 | 8 |
| 4 | hsa-miR-299 | 8 |
| 4 | hsa-miR-361 | 8 |
| 4 | hsa-miR-493 | 8 |
| 4 | hsa-miR-124 | 8 |
| 4 | hsa-miR-132 | 8 |
| 4 | hsa-miR-143 | 8 |
| 4 | hsa-miR-24 | 8 |
| 4 | hsa-miR-3163 | 8 |
| 4 | hsa-miR-34a | 8 |
| 4 | hsa-miR-34c | 8 |
| 4 | hsa-miR-449a | 8 |
| 4 | hsa-miR-449b | 8 |
| 4 | hsa-miR-498 | 8 |
| 4 | hsa-miR-512 | 8 |
| 4 | hsa-miR-520f | 8 |
| 4 | hsa-miR-577 | 8 |
| 4 | hsa-miR-588 | 8 |
| 4 | hsa-miR-654 | 8 |
| 4 | hsa-miR-656 | 8 |
| 4 | hsa-miR-877 | 8 |
